# SEVA: structural and evolutionary feature integration for predicting virulence factors and antibiotic resistance genes

**DOI:** 10.1186/s40168-026-02467-w

**Published:** 2026-07-18

**Authors:** Kaiqi Li, Xin Peng, Xiuwei Qian, Shuaicheng Li, Xianglilan Zhang

**Affiliations:** 1 Department of Computer Science, City University of Hong Kong, Hong Kong SAR, 999077 China; 2 City University of Hong Kong Shenzhen Research Institute, Shenzhen, 518057 China; 3 Department of Anaesthesia and Intensive Care, Faculty of Medicine, The Chinese University of Hong Kong, Hong Kong SAR, 999077 China; 4 Li Ka Shing Institute of Health Sciences, The Chinese University of Hong Kong, Hong Kong SAR, 999077 China; 5 Pingyu Center for Disease Control and Prevention, Zhumadian, Henan 463400 China

**Keywords:** Virulence factors, Antibiotic resistance genes, Multiple sequence alignment, Protein structure, Large protein language model

## Abstract

**Background:**

Infectious diseases continue to pose unprecedented challenges to public health and the global economy. Virulence factors (VFs) enable pathogens to adhere, reproduce, and cause damage to host cells, while antibiotic resistance genes (ARGs) enable pathogens to withstand treatments that would otherwise be effective. The concurrent identification of VFs and ARGs is crucial for efficient pathogen surveillance. However, existing tools for predicting VFs or ARGs typically suffer from high false negative rates and limitations in identifying only high-identity genes against known reference VF or ARG databases.

**Results:**

To address these challenges, we developed SEVA, an advanced model that integrates protein language models (pLMs) with structural and evolutionary protein features to predict VFs and ARGs from genome sequencing data. Integrating multiple homologous sequences can identify latent virulence or drug resistance caused by site mutations, reducing false negative rates. Meanwhile, the protein structure remains conserved despite the low sequence identity in some functional domains of VFs or ARGs. The aggregate of protein structure information further improves the identification abilities of VF and ARG. In addition, pLMs enable the model to capture high-dimensional feature representations more effectively. SEVA rigorously collected three datasets with over 20,000 genes and five reference databases. It outperforms state-of-the-art methods, including Diamond, VRprofile, FoldSeek, PreVFs-RG, PLM-ARG, ARG-BERT, and HyperVR, achieving an accuracy of 97.13% and confirming the efficacy of its key components, such as refined feature selection and multiple sequence alignment subsampling.

**Conclusion:**

SEVA takes protein sequences as input and derives evolutionary, structural, and statistical representations for prediction, making our model a reliable tool for VF and ARG prediction. This capability is particularly valuable in epidemic prevention and control, where accurate identification of VFs and ARGs is crucial. By providing concurrent and reliable predictions of VFs and ARGs, SEVA enhances our ability to respond to microbial threats effectively. This finding supports robust efforts to mitigate the spread of infectious diseases and safeguard public health, addressing a critical gap in contemporary epidemic response strategies. The SEVA model and data are available at https://github.com/kaiqili2/SEVA.

Video Abstract

**Supplementary Information:**

The online version contains supplementary material available at https://doi.org/10.1186/s40168-026-02467-w.

## Background

Virulence factor genes (VFs) and antibiotic resistance genes (ARGs) are two crucial types of genes in the microbiomes. They are commonly found in bacteria, viruses, fungi, and other microorganisms [1–4]. VFs and ARGs contribute to the development of numerous infectious diseases and pose severe challenges to human health. The mechanisms of VFs involve various pathways. Some VFs encode proteins that can destroy host cells, leading to host cell death [5]. Other VFs encode proteins known as adhesins, which enable microorganisms to adhere to the surface of host cells, providing an opportunity for invasion and infection [6]. Additionally, certain VFs interfere with the immune defenses of host, allowing microorganisms to evade host immune responses and leading to persistent infection and replication [7]. These mechanisms enable some bacterial strains to possess VFs that enhance their capacity to infect and harm their hosts.

ARGs make microorganisms resistant to antibiotics or other antibacterial drugs, preventing effective antibiotic treatments from suppressing or killing these drug-resistant strains [8]. The presence of ARGs makes microbial infections difficult to treat. With the constant evolution of the microbial landscape, new and dangerous strains of pathogens have emerged, increasing the risk of outbreaks and pandemics. Accurate identification of virulence and resistance to antibiotics can aid in understanding the pathogenesis of infections, developing vaccines, and designing targeted therapies.

With the development of high-throughput sequencing (HTS) technology, scientists are increasingly utilizing this technology to solve biological problems. Breakthroughs in high-throughput sequencing technology allow us to obtain large amounts of DNA or protein sequencing data, facilitating an in-depth understanding of the genomic information of organisms. The existing methods for predicting VFs and ARGs based on DNA or protein sequences can be generally divided into two primary categories: homology search and computational methods. Homology search methods [9, 10] consider some VFs and ARGs to be highly conserved among different organisms. The sequencing data were compared to public online VFs and ARGs databases such as VFDB [11] and CARD [12] using BLAST [13] or Diamond [14] program to find the best hit, and then sequence similarity and alignment length were calculated against known genes from these databases. VRprofile [15] enables the detection of VFs and ARGs gene clusters in bacterial genome sequences using homology searches. However, traditional homology approaches require setting specific thresholds for sequence similarity cutoff or minimum alignment length to predict potential VFs or ARGs. These methods have some limitations. First, it can only detect VFs or ARGs similar to those in the selected database, making it highly dependent on the database used. This may limit its utility in detecting new and emerging VFs or ARGs, especially those with identity less than 50% against the established databases. Second, there are no uniform standards for sequence similarity and alignment length thresholds, leading to possible inconsistencies between studies. Furthermore, these approaches often result in a high false negative rate, where actual VFs and ARGs are incorrectly identified as non-VFs or non-ARGs [16, 17]. This is a matter of concern, especially considering the rapid evolution of microorganisms and the emergence of new VFs or ARGs.

Computational methods [18–20] involve the development of prediction models based on known features and patterns associated with VFs and ARGs instead of only the best hit. PreVFs-RG [21] adopted convolutional neural networks and sequence features to predict VFs only. HyperVR [22] adopts ensemble learning by combining multiple independent machine learning models and genetic features for better prediction performance. These computational methods design predictive models by focusing on specific genetic markers and protein expression associated with drug resistance or virulence, and analyzing and extracting unique biological signatures. Nevertheless, effective feature design commonly requires substantial domain knowledge, and these features may lack generalizability across different datasets.

Large language models (LLMs) bring revolutionary changes in the field of natural language processing and have demonstrated superior performance in chatbots [23], text generation [24], and machine translation [25, 26]. Large protein language models (pLMs) consider natural amino acids as words and protein sequences arranged by amino acids as sentences. They are trained on millions of raw protein sequences to learn rich semantic protein representations from sequencing data alone. Their extraordinary feature extraction capabilities have succeeded in many aspects of protein-related tasks, such as protein function prediction, protein structure prediction, and protein design. Unlike traditional methods that rely on explicit homology, pLMs are trained in a self-supervised manner on millions of diverse protein sequences, enabling them to learn a high-dimensional, contextual understanding of protein semantics. Furthermore, pLMs eliminate the need for manual feature engineering driven by domain experts. The embeddings they generate serve as powerful, general features that capture the functional properties of proteins. ESM [27] has trained the Transformer architecture on 250 million protein sequences of the Uniparc database for learning biological properties and has shown remarkable performance on secondary structure, tertiary contacts, and mutational effect. ProtT5-XL-U50 [28] has trained the T5 architecture on UniRef50 database to capture biophysics features of protein sequences. MSA Transformer [29] has trained the 12-layer Transformer architecture on a dataset of 26 million Multiple sequence alignments (MSAs). pLM-based models can learn protein-related feature representations for predicting VFs and ARGs, such as PLM-ARG [30], ARG-BERT [31], VirulentHunter [32], and the work by Malekian [33].

Moreover, the accurate protein structure prediction from amino acid sequences has been revolutionized by methods such as AlphaFold2 [34]. A critical aspect of its architecture is the integration of evolutionary information through MSAs, a fundamental bioinformatics technique that identifies conserved and co-evolving residues across homologous sequences. These patterns of conservation provide essential constraints for inferring structural contacts. The high-accuracy structural information serves as a primary source of features for predicting protein function. FoldSeek [35] is a fast and accurate software tool for comparing protein structures and identifying similar proteins at the structural level, even when their amino acid sequences are vastly different. Structural information is considered to be more reflective of protein function than sequences.

As seen above, numerous approaches have been devised for predicting and analyzing VFs and ARGs, and progress has been made in improving their predictive performance. However, several issues still need to be addressed in these approaches: (1) The majority of these tools tend to produce a high rate of false negatives [16, 17], particularly for genes with low identity compared to the known VFs or ARGs databases, suggesting a need for a more robust, generalizable model. (2) Current methods do not completely exploit feature representation capabilities. Potential remains in how these methods can automatically learn and extract meaningful features and patterns directly from raw sequencing data for improving prediction accuracy. (3) Interactions between features are lacking or quite limited. Existing algorithms learn each feature representation independently in their separate encoder, and mutual information is integrated only at the decoder stage [36, 37]. This isolation hinders a comprehensive understanding of the patterns and relationships between different features, which is crucial for prediction accuracy.

Here, we introduce SEVA, a deep learning approach that integrates *S*tructural and *E*volutionary features to predict *V*irulence factors and *A*ntibiotic resistance genes from genome sequencing data and aggregates large protein language models to capture rich feature representations. Specifically, we first generate MSAs of the target protein sequence by searching genetic databases. These MSAs are then used as input to AlphaFold2 to predict their three-dimensional protein structures. MSA Transformer, a large protein language model pre-trained on millions of diverse sequences to capture feature representations, was employed in its original form to generate the MSA embeddings used in SEVA. Additionally, the 3D protein structure predicted by AlphaFold2 was employed to calculate the residue distance maps as structural features. The structural and evolutionary feature representations are subsequently fed into the feature fusion module designed to learn joint interaction representations, thereby facilitating a deeper, more accurate predictive process. MSA of different homologs revealed evolutionarily conserved positions that often correspond to functionally critical sites, enabling SEVA to detect distant homology missed by traditional methods. This feature is critical for accurately predicting the functions of rapidly evolving VFs and ARGs while reducing false positives through evolutionary constraint analysis. The combination of these evolutionary features with structural information further improves predictive reliability for pathogen surveillance. We collected three datasets with more than 20,000 genes and five reference databases. The performance of SEVA was evaluated using the test dataset consisting of 209 VFs, 209 ARGs, and 209 negative samples (NSs). The results revealed that SEVA outperforms contemporary state-of-the-art tools such as Diamond, VRprofile, FoldSeek, PreVFs-RG, PLM-ARG, ARG-BERT, and HyperVR, achieving an accuracy of 97.13%. To further assess the generalization ability of SEVA, we evaluated the model on a low-identity benchmark dataset and simulated bloodstream infection metagenomic samples. Because the full SEVA pipeline requires MSA construction and structure prediction, we also provide SEVA-Fast, a lightweight mode for rapid large-scale screening when computational efficiency is prioritized.

**Fig. 1:**
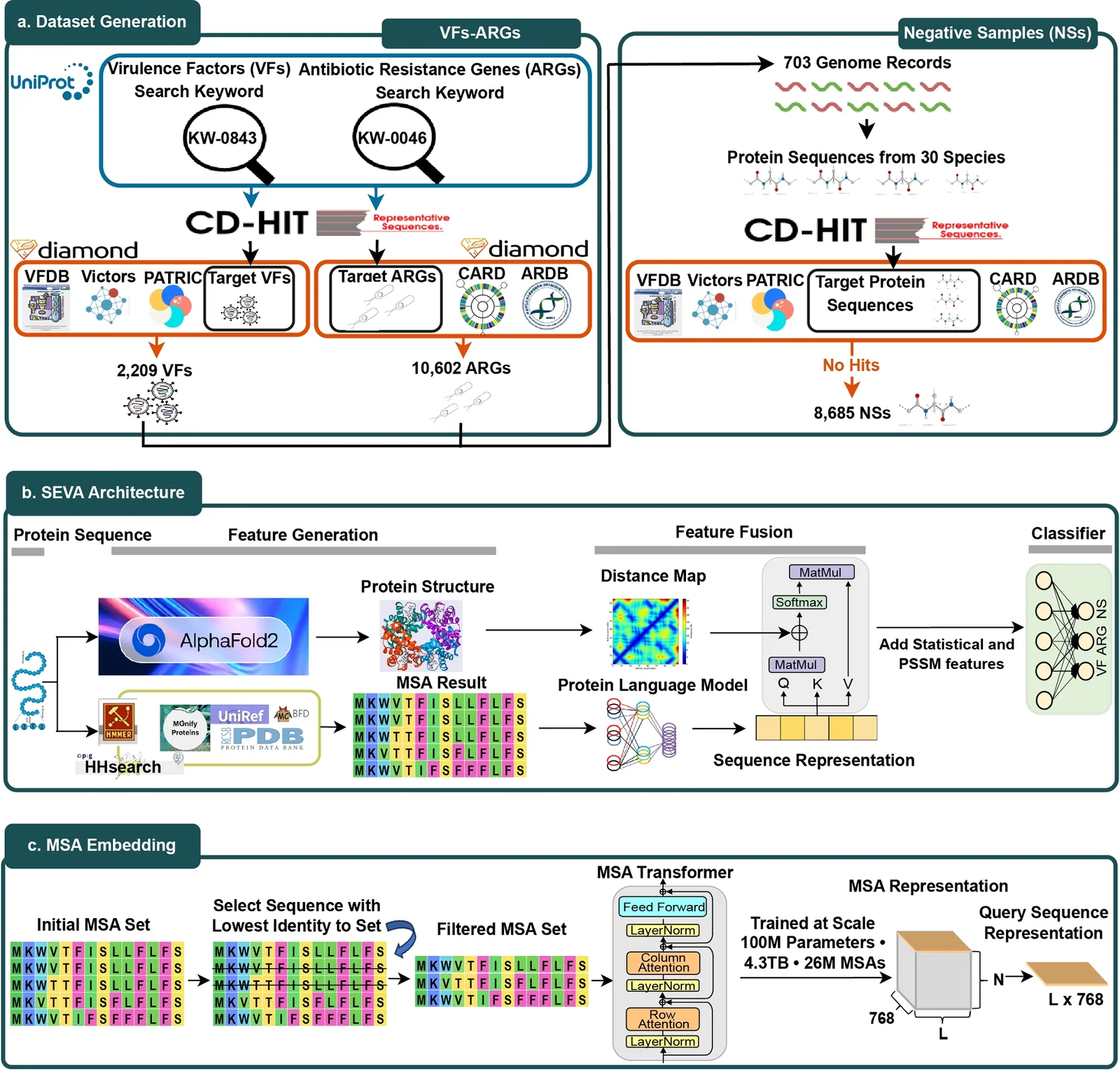
Overview of the SEVA architecture. **a** Workflow for constructing the VF, ARG, and NS datasets. VFs and ARG are collected from UniProt (keyword: KW-0843 and KW-0046), with redundancy and low-quality data filtered out using CD-HIT and Diamond. NSs are constructed from 30 representative species, retaining only those protein sequences that showed no match to the reference databases. **b** With a protein sequence as input, the MSA is generated by genetic database search and protein structure is predicted by AlphaFold2. The distance map is calculated according to the protein structure, which encodes the spatial distance between residues of the input protein sequence. The pre-trained language model MSA Transformer is used to obtain MSA embedding. The MSA embedding and distance map are fed into the feature fusion module to learn local feature interactions, which are then aggregated with statistical and PSSM features into the classifier for predicting VFs, ARGs, and NSs. **c** MSA embedding strategy. The maximum diversity strategy is used to filter high-confidence similarity sequences in MSA. The MSA feature representation generated by MSA Transformer only selects the query sequence representation

## Methods

### SEVA model

The SEVA model, implemented in the PyTorch [38] framework, was designed to identify virulence factor genes and antibiotic resistance genes. As shown in Fig. 1, protein sequences were used as input. SEVA takes protein sequences as input and employs structural and evolutionary representations for model training and inference. The excellent protein structure prediction model AlphaFold2 was used to generate the 3D protein structure with default parameters. In this process, five protein structures could be predicted for a given protein sequence, each corresponding to one of the five unique model parameters provided by AlphaFold2. This approach enhances the prediction quality. The distance maps $D_{ij} \in \mathbb {R}^{L\times L\times 6}$ were calculated from protein structure representations, where *L* is the length of the protein sequence. Five distance maps were first generated from the five AlphaFold2-predicted structures, and the sixth map was calculated as the average of the first five maps.

1 $$\begin{aligned} D_{ij} = \frac{1}{ 1 + d_{ij}}, \end{aligned}$$

where $d_{ij}$ describes the spatial distance between residue *i* and *j*.

The MSA is generated by searching protein databases such as Uniref [39], MGnify [40], BFD [41], and PDB [42] using JackHMMER [43] and HHsearch [44]. All hits were filtered with an E-value cutoff of 0.001 and a minimum coverage threshold of 50% to ensure high-quality alignments. The computational and memory costs positively correlate with the number of MSAs, and the original MSAs generally include thousands of protein sequences. It is necessary to decrease the number of MSA features. We employed the maximum diversity strategy to subsample 256 sequences in MSA for computational reasons. The maximum diversity strategy is a greedy algorithm that selects the protein sequence with the lowest identity against the current set of sequences, thereby reducing redundancy and maintaining maximum diversity. The maximum diversity MSA selection algorithm is illustrated in Supplementary file 1: Fig. S1. Diverse MSAs can cover a broader range of protein sequence variation, which is essential for understanding protein function.

To aggregate evolutionary and structural information, SEVA used the residue-level MSA embedding as the main input to the multi-head self-attention module [45] and incorporates the structure-derived distance map into the attention calculation. Specifically, we utilized the protein language model MSA Transformer to encode the MSA feature. MSA Transformer inputs MSA, a 256×L character matrix, and outputs $256 \times L \times 768$ tensors. We extracted the embedding of the query sequence, denoted as $M \in \mathbb {R}^{L\times 768}$, where *L* is the sequence length. For each attention head, the query, key, and value matrices are generated as:

2 $$\begin{aligned} Q_h = MW_h^{Q}, K_h = MW_h^{K}, V_h = MW_h^{V} \end{aligned}$$

where $W_h^{Q}$, $W_h^{K}$, and $W_h^{V}$ are learnable projection parameter matrices, *h* is the number of heads. The residue distance map *D* calculated from the AlphaFold2-predicted structure is then incorporated directly into the self-attention calculation:

3 $$\begin{aligned} A_h = softmax\left( \frac{Q_hK_h^{T}}{\sqrt{d_k}} + D\right) \end{aligned}$$

4 $$\begin{aligned} Head_h = A_hV_h \end{aligned}$$

5 $$\begin{aligned} MultiHead(M,D) = Concat(head_1,...,head_h)W^{O} \end{aligned}$$

where $d_k$ is the dimension of the feature representation of the query, $A_h$ is the attention weight matrix. In our work, we employed $d_k$ = 128, *h* = 6. Thus, the MSA embedding provides an evolutionary sequence representation, while the structural feature is introduced as a term that constrains residue-residue attention based on spatial proximity. This design enables SEVA to learn joint representations in which residues that are both evolutionarily informative and structurally close can receive high attention scores. The multi-head self-attention concatenates the results of all six heads and projects them with parameter $W^{O}\in \mathbb {R}^{768\times d_k}$. The multi-head self-attention block is stacked twice in our work to capture and integrate complex features effectively. To identify the optimal model architecture, we performed ablation studies on the hyperparameters of SEVA. The configuration achieving the best performance comprised two attention layers with six heads each and a dropout rate of 0.2 (Supplementary file 1: Fig. S7).

Finally, the results of the Transformer encoder are fed into a multi-layer perceptron to generate the prediction. In addition, some statistical and position-specific scoring matrix (PSSM) features were integrated into the classification module. Statistical features include amino acid composition (AAC) [46], dipeptide composition (DPC) [47], dipeptide deviation from expected mean (DDE) [47], pseudo-amino acid composition (PAAC) [48, 49], and quasi-sequence order (QSO) [50, 51]. PSSM is a profile representation of an MSA that assigns position-dependent scores to amino acids based on their observed conservation and variation at each aligned residue position. In our study, PSSM was directly generated using the PSI-BLAST program [13] with an MSA as input. Three PSSM features were then extracted: PSSM composition [52], RPM-PSSM [53], and AADP-PSSM [54]. Detailed information on statistical and PSSM features is provided in Supplementary file 1: Table S2. The cross-entropy loss function was applied to train our model.

6 $$\begin{aligned} L_{CE} = -\sum \limits _{i} y_i \log (p_i) \quad (i=1,2,3) \end{aligned}$$

where $y_i$ is the target label, and $p_i$ is the prediction probability. The i=1,2,3 represent the VF, ARG, and NS, respectively.

### Datasets

#### Training and test datasets

In this study, we collected protein sequences related to virulence factors, antibiotic resistance genes, and negative samples. The data process workflow is shown in Fig. 1a. For VFs, the research sourced data from four databases: PATRIC [55], Victors [56], VFDB [11], and UniProt [57]. Following the previous work [20, 22], we first retrieved VF protein sequences with the query “keyword: ‘Virulence [KW-0843]’ ” in Uniprot databases and employed PATRIC, Victors, and VFDB as reference databases to ensure VF quality. The CD-HIT [58] program was adopted to cluster these sequences from these databases, aiming to eliminate duplicates or highly identical data with hyperparameters set for 100% identity. In this way, 2209 VFs remained in the UniProt database.

For ARGs, the research sourced data from three public databases: CARD [12], ARDB [59], and UniProt [57]. Similar to the VFs collection process, we retrieved ARG protein sequences with the query “keyword: ‘Antibiotic resistance [KW-0046]’ ” in Uniprot databases, and processed all selected genes through CD-HIT with 100% identity parameters and compared them to the CARD and ARDB databases for removing redundant and low-quality data. Finally, 10,602 ARGs in Uniprot were collected for downstream analysis.

For NSs, defined here as genes that are neither VFs nor ARGs, we constructed a dataset designed to better capture true protein distribution and diversity. Starting from available well-annotated and complete genomes containing VFs and ARGs, we first removed duplicate entries, leaving 703 distinct genome records. From these, 30 representative species were selected, and their complete protein sequences were downloaded (see Supplementary file 1: Table S8). To ensure data quality, annotations corresponding to unknown, hypothetical proteins, VF, or ARG were filtered out. Protein sequences with no matches to reference databases (VFDB, Victors, PATRIC, CARD, ARDB, VF, and ARG) were retained as NSs, resulting in 8685 sequences.

To reduce sequence redundancy and avoid overlap between training and test sets, VF, ARG, and NS sequences were separately clustered using CD-HIT at a 90% sequence identity threshold. The dataset was then split at the cluster level: all sequences within the same CD-HIT cluster were assigned to either the training or test set. Thus, no CD-HIT cluster contributed sequences to both training and test datasets. For VFs, 209 sequences from different clusters were selected as the test set, and sequences from the remaining clusters were used for training. The same cluster-level splitting procedure was applied to ARGs and NSs. To balance the dataset, we sampled 209 ARGs and 209 NSs from distinct clusters for the test set, and 2000 ARGs and 2000 NSs for the training set. This procedure yielded a training dataset of 6000 sequences (2000 per category) and a test dataset of 627 sequences (209 per category). We also conducted experiments using additional CD-HIT identity thresholds (0.4, 0.5, 0.6, 0.7, 0.8, and 1.0; Supplementary file 1: Table S6), which demonstrated that SEVA consistently achieved an overall accuracy above 94.90%.

Moreover, to maximize the utility of the available data, we created a new and more challenging independent benchmark dataset for ARG detection by incorporating 8393 sequences that had been set aside during the initial dataset balancing (derived from a total of 10,602 sequences, minus 2209 used for training and testing). To ensure independence and reduce redundancy, we applied homology filtering using CD-HIT with a 90% sequence identity threshold against the training set. This process yielded a test set consisting of 3684 non-redundant ARG sequences.

#### Low-identity dataset

To establish a robust benchmark to evaluate the ability of our model to detect evolutionarily distant sequences, we developed a carefully curated low-identity dataset through a multi-stage validation process. VF candidate data from UniProt Swiss-Prot database and VirulentHunter [32]. ARG candidate data from UniProt Swiss-Prot database and HMD-ARG [60]. NS candidate data from previously selected 30 species genome protein sequences. These candidates were then subjected to rigorous sequence validation against specialized reference databases (PATRIC, Victors, VFDB, and training VFs data for virulence factors; CARD, ARDB, and training ARGs data for antibiotic resistance genes; training NSs data for negative samples) using CD-HIT with a 50% sequence identity cutoff. The resulting dataset of 1475 VFs, 1605 ARGs, and 1500 NSs, all with less than 50% identity to our training sequences and reference databases, represents a challenging test case for VF and ARG prediction.

#### Simulated bloodstream infection dataset

To simulate real bloodstream infection (BSI) samples, we introduce the microbial community standard into mouse blood. The mock microbial community consists of eight bacterial and two fungal strains. Their theoretical composition, based on genomic DNA, is as follows: *Listeria monocytogenes* at 89.1%, *Pseudomonas aeruginosa* at 8.9%, *Bacillus subtilis* at 0.89%, *Saccharomyces cerevisiae* at 0.89%, *Escherichia coli* at 0.089%, *Salmonella enterica* at 0.089%, *Lactobacillus fermentum* at 0.0089%, *Enterococcus faecalis* at 0.00089%, *Cryptococcus neoformans* at 0.00089%, and *Staphylococcus aureus* at 0.000089%. To ensure uniformity in experimental materials, we pooled and gently mixed all collected mouse blood in a single tube before aliquoting into 1.5 mL centrifuge tubes. We then retrieved the ZymoBIOMICS Microbial Community Standard II (D6310, batch number: 210560) from a − 80$^{\circ }$C freezer, vortexed it for thorough homogenization, and diluted it in PBS buffer to targeted concentrations of $1.5 \times 10^3$, $1.5 \times 10^4$, and $1.5 \times 10^5$ cells/μL. For each simulation, we combined 10 μL of the precisely diluted microbial standard with 40 μL of PBS buffer and 350 μL of mouse blood to fabricate a simulated BSI sample.

Subsequent sequencing of the BSI samples was performed using the Illumina NextSeq 2000 system. For DNA extraction, we employed the High Pure PCR Template Preparation Kit. Library construction was conducted using the QIAseq FX DNA Library Kit, with library fragment sizes controlled between 300 and 400 bp. Following quantification with the Qubit 4.0 Fluorometer, immediate quality control of the libraries was carried out. For library quality assessment, we utilized the Qsep100 bio-fragment analyzer. The analysis parameters were set as follows: the sample voltage at 4 kV, sampling time at 10 s, separation voltage at 6 kV, and separation time at 300 s. Using the standard S2 cartridge, libraries displaying a relative fluorescence intensity $\ge \!0.5$ were considered of high quality, while those with an intensity <0.5 were classified as low quality. The selected high-quality libraries were subsequently sequenced on the Illumina NextSeq 2000 platform using a metagenomic sequencing protocol with a single-end SE100 configuration by producing reads of 100 bp.

The raw reads obtained from the sequencing platforms served as the initial data, which were subjected to quality control using fastp [61]. We then used MEGAHIT [62] to assemble the processed fastq files to generate contigs sequences. Subsequently, we used seqGraph to connect and extend these contigs. Finally, we annotated the extended assembly results with prokka [63] to obtain predicted genes and corresponding protein predictions on the contigs. All other parameters not specifically listed were set to the default values provided by the respective software tools. In this process, we collected 15,097 sequences for analysis.

The labels of the simulated BSI dataset were determined through database annotation at the sequence or structure level rather than experimental validation. Specifically, protein sequences predicted from the assembled contigs were compared with VF and ARG reference databases (VFDB, Victors, PATRIC, CARD, and ARDB). Proteins with sequence identity $\ge \!80\%$ and alignment coverage $\ge \!80\%$ to reference databases were treated as sequence-based annotated VF/ARG. In addition, AlphaFold2-predicted protein structures were compared with known VF/ARG reference structures using FoldSeek, and proteins satisfying the probability threshold $\ge \!0.5$ were treated as structure-based annotated VF/ARG. Proteins supported by either sequence-based or structure-based annotation were labeled as VF/ARG targets. Finally, 863 sequences were labeled as VF, and 128 sequences were labeled as ARG. These annotations served as the reference labels for model evaluation on the simulated BSI dataset.

### Model training and implementation

The deep learning network of SEVA was implemented on Python 3.8 with PyTorch backend. Model training was accelerated using an NVIDIA V100 GPU with 32 GB GPU memory. The network was optimized using the Adaptive Moment Estimation (Adam) algorithm [64] with a learning rate of 0.0001. Training was performed using a batch size of 32 across 50 epochs. To enhance generalization and prevent overfitting, we incorporated several regularization strategies: a dropout rate of 0.2 and L2 regularization (weight decay of 0.00001). Furthermore, an early stopping strategy was employed. The model began to converge around 20 epochs and stabilized around 35.

### Performance metrics

In order to evaluate the SEVA model, our study employed four recognized multiclass standard performance metrics to ensure a comprehensive and accurate performance assessment. The key metrics include precision, recall, and the F1-score, along with their micro-averages.

7 $$\begin{aligned} Precision_i = \frac{TP_i}{TP_i + FP_i} \quad (i = 1, 2, 3), \end{aligned}$$

8 $$\begin{aligned} Recall_i = \frac{TP_i}{TP_i + FN_i} \quad (i = 1, 2, 3), \end{aligned}$$

9 $$\begin{aligned} F_1-score_i = \frac{2 \times Precision_i \times Recall_i}{Precision_i + Recall_i} \quad (i = 1, 2, 3), \end{aligned}$$

10 $$\begin{aligned} Accuracy = \frac{\sum TP_i}{N} \quad (i = 1, 2, 3), \end{aligned}$$

True positives (TP), false positives (FP), true negatives (TN), and false negatives (FN). These components play a crucial role in quantifying the performance of the model. TP refers to accurately identifying a sample as either an ARG, a VF, or an NS. FP indicates a sample as belonging to one of the categories (VF, ARG, NS) when it does not actually belong to that category. TN indicates the ability of the model to recognize samples that do not belong to any of the specified categories (VF, ARG, NS). FN means the number of samples that the model fails to predict. *N* is the total number of samples. In this evaluation, the categories are as follows: *i* = 1 for VF, *i* = 2 for ARG, and *i* = 3 for NS. These classification indicators form the backbone of the performance metrics, enabling a comprehensive analysis of the SEVA model’s effectiveness.

## Results

### The SEVA architecture

We developed a deep learning-based model, SEVA, using the Transformer architecture to aggregate protein structural and evolutionary information for accurately predicting VFs and ARGs. A more detailed description of our model is provided in the “Methods” section, and the overview of SEVA is shown in Fig. 1.

Given a protein sequence, MSA should be generated by genetic database searching and protein structure should be predicted by AlphaFold2. SEVA aggregates structural and evolutionary features using Transformer architecture to capture protein-related representations. Evolutionary features are fed into the Transformer to produce the attention map, which learns similarities between each protein sequence. Moreover, to consider spatial structure information in our method, we employ the distance map obtained from protein structure to further constrain the attention map. In this process, SEVA simultaneously aggregates sequence-based features and spatial structure features to learn complex feature representations, which are used to predict VFs, ARGs, and NSs in the classifier. Additionally, the computational and memory cost of the MSA module is positively related to the depth of MSA [65], and the original MSA generally contains thousands of homology sequences. Thus, it is highly desirable to decrease the depth of MSA, considering the limited computational resources. We employ the MSA selection strategy to filter the sequence with high identity against the current MSA set to maximize the diversity of the MSA (Fig. 1c).

SEVA differs from previous works in three major aspects. Firstly, SEVA integrates structural and evolutionary features to predict VFs and ARGs from genome sequencing data. Key residues are often conserved across homologs, even in distantly related proteins. MSA columns with high conservation indicate functionally critical regions. Aggregating MSA features aims to build a more robust and generalizable model that can reduce the rate of false negatives and improve predictive performance, especially for genes with low identity compared to known databases. Secondly, SEVA utilizes the MSA Transformer, a pre-trained protein language model that has been exposed to millions of diverse sequences. This allows for the generation of complex MSA embeddings, which leads to an advanced understanding of protein sequences. The deployment of a large and potent protein language model represents a significant advancement, enhancing the discriminative ability of the model to adaptively extract and distill previously unidentified yet crucial and significant features and patterns from raw sequencing data. Thirdly, the approach includes a feature fusion module designed to learn joint interaction representations. Instead of learning each feature representation independently, this module allows for the mutual information of different features to be integrated earlier in the process. This facilitates a deeper and more accurate predictive process by providing a comprehensive understanding of the patterns and relationships among different features.

**Fig. 2:**
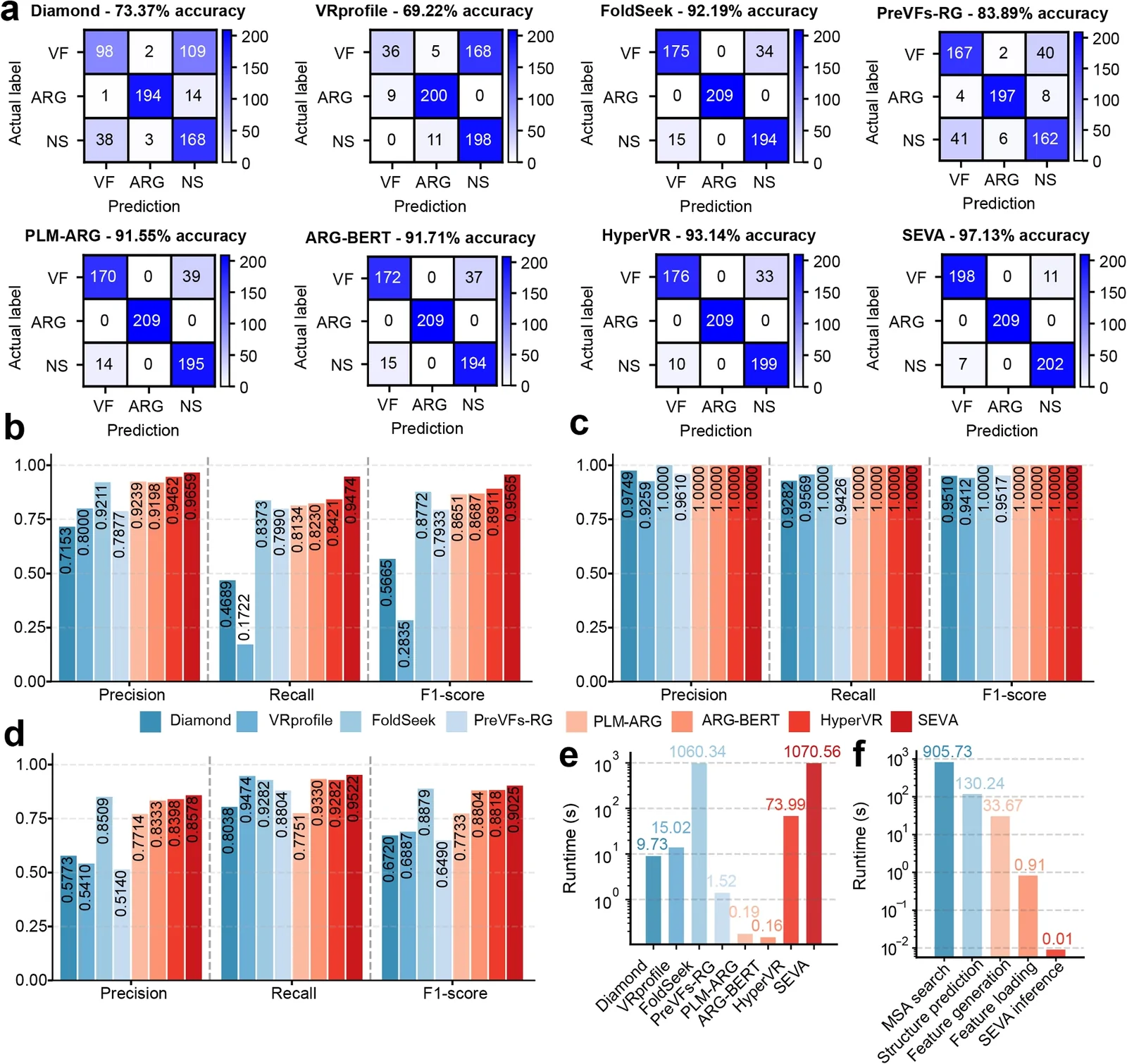
The performance of seven existing models and the proposed SEVA on concurrent prediction of VFs, ARGs, and NSs. **a** Confusion matrix comparing prediction accuracy across eight tools, including Diamond, VRprofile, FoldSeek, PreVFs-RG, PLM-ARG, ARG-BERT, HyperVR, and SEVA, respectively. **b** The precision, recall, and F1-score of these eight tools to predict VFs. **c** The precision, recall, and F1-score of these eight tools to predict ARGs. **d** The precision, recall, and F1-score of these eight tools to predict NSs. **e** Runtime comparison of the eight tools. **f** Runtime of individual SEVA components. MSA search, structure prediction, feature generation, and feature loading all contribute to feature preparation, whereas SEVA inference alone takes less than 1 second per sequence

### SEVA outperforms the state-of-the-art methods

To evaluate the performance of SEVA, we compared the proposed model with existing tools, such as Diamond [14], VRprofile [15], FoldSeek [35], PreVFs-RG [21], PLM-ARG [30], ARG-BERT [31], and HyperVR [22]. To prevent potential information leakage during MSA generation, strict identity-based procedures should be implemented. For the construction of the MSA for the test dataset, similar to the dataset splitting threshold, sequences in the MSAs that are more than 90% identity against any protein in MSAs in the training data were removed. The performance of these methods is illustrated in Fig. 2.

Diamond and VRprofile are sequence homology search approaches, and both need a choice of thresholds. The performance of the Diamond homology search method with different identity thresholds (0.3, 0.4, 0.5, 0.6, 0.7, 0.8) is shown in Supplementary file 1: Fig. S2. We utilize the threshold (0.3) that maximizes accuracy to fairly compare with other tools, and the comparison databases including VFDB, Victors, PATRIC, CARD, and ARDB. For VRprofile, we kept the default parameters to run the programs. Diamond and VRprofile achieved comparable results in predicting ARGs but differed in predicting VFs, likely due to differences in the composition and coverage of their respective reference databases. This difference highlights a fundamental limitation of sequence-based methods: their dependence on reference databases that may not cover all protein variants or novel, uncharacterized virulence factors and ARGs [13, 14]. In addition, PathoFact [17] achieves 71.45% accuracy on the test dataset when toxin predictions are ignored from the evaluation (Supplementary file 1: Fig. S9).

FoldSeek, a structure-aware alignment tool, demonstrated excellent performance, with its accuracy peaking at 92.19% using a probability threshold of 0.5 (Supplementary file 1: Fig. S3). For the FoldSeek analysis, we constructed a custom structure database comprising proteins from VFDB, Victors, PATRIC, CARD, and ARDB. Query structures were predicted using AlphaFold2. Searches were performed using the easy-search mode with default parameters. This highlights the critical importance of integrating structural information for the accurate prediction of VFs and ARGs. For a direct comparison, we evaluated SEVA using only its structural component. SEVA with structure features only attained 92.50% accuracy (Supplementary file 1: Table S7), performing on par with the specialized FoldSeek tool. The result demonstrates that SEVA effectively learns discriminative structural features and patterns.

For machine learning based approaches, we trained these models on our training data for fair comparison. PreVFs-RG and HyperVR are feature-based approaches and use classical machine learning algorithms to predict VFs and ARGs by manually extracting biological features. The performance of feature-based approaches is better than sequence homology search methods. In addition, HyperVR performs better than PreVF-RG because HyperVR incorporates evolutionary information. PLM-ARG and ARG-BERT using pLMs to learn latent protein sequence representations. These PLM-based models obtain relatively good performance. Simply obtaining high-dimensional features from sequences is not enough.

SEVA achieved an overall accuracy of 97.13%, outperforming Diamond, VRprofile, FoldSeek, PreVFs-RG, PLM-ARG, ARG-BERT, and HyperVR by 23.76%, 27.91%, 4.94%, 13.24%, 5.58%, 5.42%, and 3.99%, respectively. SEVA adopts a more efficient and powerful algorithm with excellent semantic modeling and feature expression capabilities. In addition, the features extracted by SEVA are richer, and structural and evolutionary features can enhance the ability of the model to learn homology information and explain complex patterns in biological data. SEVA appears to be based on a more flexible approach that considers multiple biological features and large protein language models and may be better equipped to identify novel VFs and ARGs. Moreover, the comparative performance on machine learning based methods under the five-fold cross-validation process further validates the generalization capabilities and robustness of the SEVA model (Supplementary file 1: Table S4).

To further evaluate SEVA’s performance in ARG detection, we used an independent ARG test dataset for comparative evaluation. The results are shown in Supplementary file 1: Fig. S10. SEVA achieved the highest recall of 98.59%, improving over Diamond, VRprofile, FoldSeek, PreVFs-RG, PLM-ARG, ARG-BERT, and HyperVR by 2.31%, 1.79%, 0.90%, 1.98%, 1.01%, 0.84%, and 0.57%, respectively. SEVA also obtained the best F1-score of 99.29%; compared with the same set of baselines, the corresponding gains were 1.32%, 1.13%, 0.46%, 1.12%, 0.51%, 0.43%, and 0.29%. For precision, SEVA reached 1.0000, the same as FoldSeek, PLM-ARG, ARG-BERT, and HyperVR, and slightly above Diamond, VRprofile, and PreVFs-RG by 0.28%, 0.45%, and 0.22%, respectively.

The runtime of the prediction process between the above eight tools was compared. To ensure a fair comparison of computational efficiency, all tools were benchmarked on an identical hardware configuration utilizing a single Intel(R) Xeon(R) Platinum 8280 CPU, 64 GB CPU RAM, and a single Tesla V100 GPU with 32 GB GPU memory. Figure 2e shows the runtime of each SEVA component on the test dataset. PLM-ARG and ARG-BERT (less than 0.2 s per sequence) are fast tools to identify VFs and ARGs using the representation ability of pLMs, but they are not as good as SEVA in prediction accuracy. Structure-aware approaches required substantially longer runtimes than the other evaluated tools, with FoldSeek and full SEVA requiring 1060 and 1070 s per sequence, respectively. MSA search and structure prediction processes constitute the majority of the runtime of SEVA. When MSA and structural features have already been generated and stored, SEVA inference requires less than 1 second per sequence. However, for newly submitted sequences, MSA search and structure prediction remain the main computational bottlenecks of the full SEVA pipeline. To improve computational efficiency, we developed a fast mode of SEVA (SEVA-Fast), which omits the computationally intensive MSA search and structural prediction steps and instead relies on statistical features such as AAC, DPC, DDE, PAAC, and QSO. SEVA-Fast requires 0.36 s per sequence and achieves 91.23% accuracy on the test dataset (Supplementary file 1: Table S9). Thus, SEVA provides a practical speed-accuracy trade-off: users can select the full SEVA model for maximum predictive performance, or use SEVA-Fast for high-throughput screening with substantially reduced runtime but relatively lower accuracy. Both variants are available in the SEVA software package.

In addition, to better understand the discriminative power of SEVA, we analyze the identity distribution of VFs from the test dataset against the training dataset. The result shows that the identity of all misclassified VFs is less than 40% compared with the training dataset, and 4/7 misclassified VFs have an identity of less than 10% (Supplementary file 1: Fig. S4). The discriminability of low-identity data has always been a major concern in predicting VFs and ARGs [30, 32]. SEVA maintains robust performance across a wide range of sequence identity, but still encounters limitations in low-identity data.

### Structural and evolutionary features enhance the discriminative ability

**Fig. 3:**
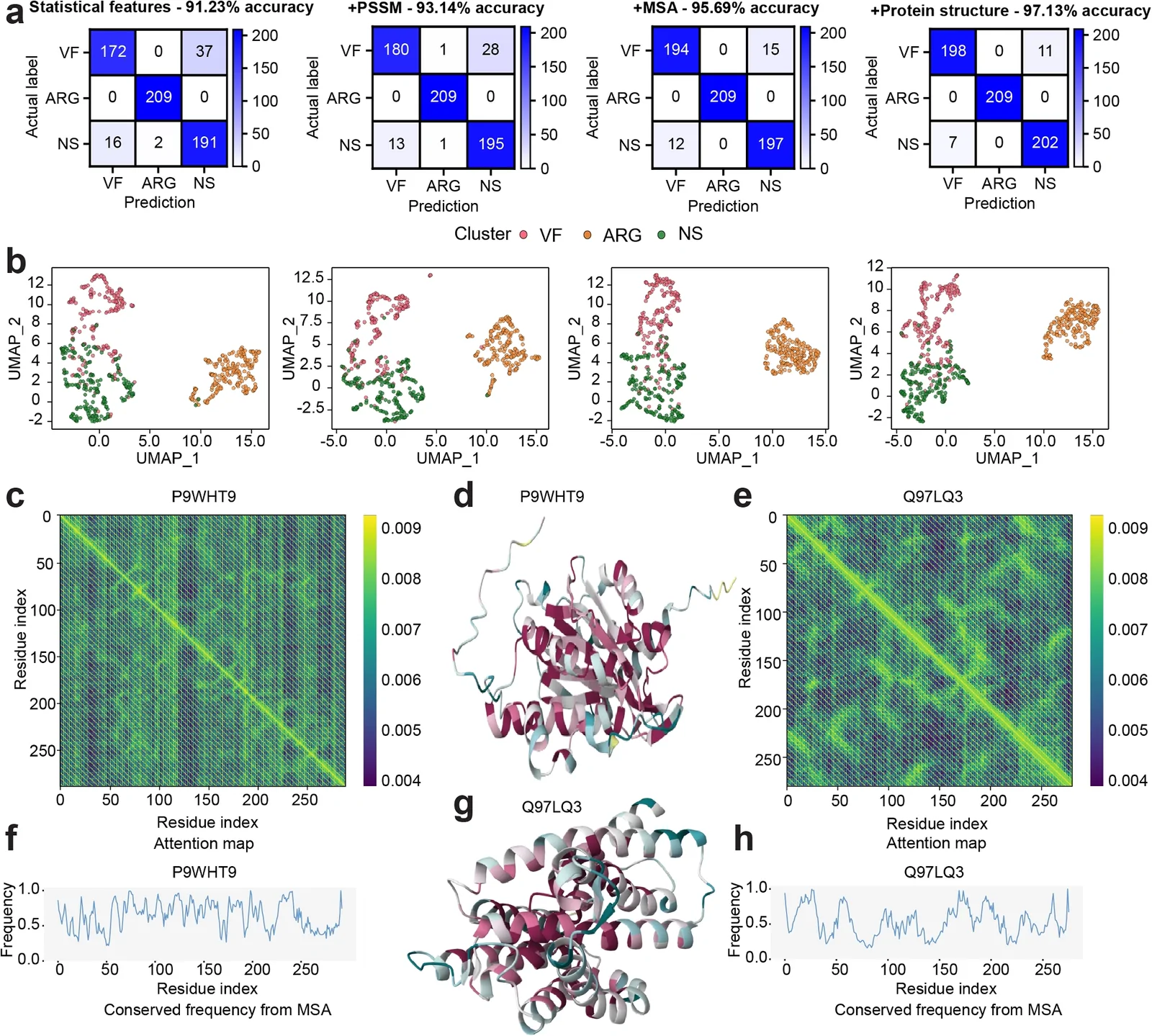
Comparison with different biological features and visualization analysis of P9WHT9 and Q97LQ3. **a** Confusion matrices comparing cumulative prediction accuracy across different features, including statistical features, PSSM, MSA, and protein structure, respectively. **b** UMAP to cluster the sequences based on their SEVA outputs. It shows the cumulative results of statistical features, PSSM, MSA, and protein structure from left to right. **c** The attention map between the residues of P9WHT9. **d** Attention values mapped onto the protein structure of P9WHT9, residues with high attention values are highlighted in dark pink. **e** The attention map between the residues of Q97LQ3. **f** Smoothed conserved frequency from MSA of P9WHT9. **g** Attention values mapped onto the protein structure of Q97LQ3, residues with high attention values are highlighted in dark pink. **h** Smoothed conserved frequency from MSA of Q97LQ3

The comparative evaluation of diverse features provides valuable insights into their contributions to VF and ARG prediction performance, guiding the selection and integration of features in future model development efforts. By evaluating the impact of different feature types, we understand how each feature influences the ability of the model to discriminate between VFs and ARGs. In our study, we conducted a series of experiments to evaluate the performance of gene prediction models incorporating various features: statistical features, position-specific scoring matrices, multiple sequence alignment, and protein structural information derived from AlphaFold2. Detailed information on statistical features and PSSM is shown in Supplementary file 1: Table S2. The cumulative performance of these diverse features is comprehensively detailed in Table 1 and Fig. 3.

**Table 1:** The cumulative performance of the proposed model with different biological features

Features	Gene	Precision	Recall	F1-score	Accuracy
**Statistical features**	VF	0.9149	0.8230	0.8665	0.9123
	ARG	0.9905	1.0000	0.9952	
	NS	0.8377	0.9139	0.8741	
**+PSSM**	VF	0.9326	0.8612	0.8955	0.9314
	ARG	0.9905	1.0000	0.9952	
	NS	0.8744	0.9330	0.9028	
**+MSA**	VF	0.9417	0.9282	0.9349	0.9569
	ARG	1.0000	1.0000	1.0000	
	NS	0.9292	0.9426	0.9359	
**+Protein structure**	VF	0.9659	0.9474	0.9565	0.9713
	ARG	1.0000	1.0000	1.0000	
	NS	0.9484	0.9665	0.9573	

Table 1 shows excellent performance in VFs and ARGs prediction when these features are included in the computational model. When using only Statistical features, the average accuracy is 91.23%. Upon incorporating PSSM, accuracy increases by 1.91%. Adding MSA on top of the previous two features further boosts accuracy to 95.69%, making a 2.55% improvement over the previous iteration. Finally, when utilizing all four features, including Statistical features, PSSM, MSA, and Protein Structure, the proposed model achieves an overall accuracy of 97.13%. Additionally, to visualize the results of SEVA with different features, we employed uniform manifold approximation and projection (UMAP) to explore how these features contribute to the performance of the SEVA model. As shown in Fig. 3b, clusters become clearer when these features are incorporated in SEVA, especially MSA and protein structure features. SEVA’s ability to integrate and effectively utilize these features could be a key factor in its improved performance, providing a more comprehensive analysis than methods relying on primary sequencing data or basic statistical features.

To further investigate the ability of SEVA to capture biologically meaningful patterns, we performed attention analysis by extracting attention weights from the trained SEVA model during inference and visualizing them as attention maps for a representative VF (UniProt ID: P9WHT9) and an ARG (UniProt ID: Q97LQ3). The attention weights correspond to $A_h$ in Eq. 3. As shown in Fig. 3c and e, the attention maps reveal residue-residue interaction patterns for each protein. To evaluate whether these attention weights reflect evolutionary and structural features, we compared the attention highlights with the residue conservation profile derived from the MSA of each protein (Fig. 3f and h). We observed a clear correspondence between regions of high attention in the model and positions with high conservation in the MSA, suggesting that the model effectively learns evolutionary information. Furthermore, we projected the attention weights onto the 3D structures of both proteins. Residues with high attention values are highlighted in dark pink in the structural visualization (Fig. 3d and g). These highly attentive residues are often in close proximity within the folded protein, indicating that the model captures not only sequential co-evolution but also structural constraints. These results demonstrate that the attention mechanism in SEVA successfully encodes both MSA and 3D protein structure relationships.

### Maximum diversity improves MSA embedding for SEVA

**Fig. 4:**
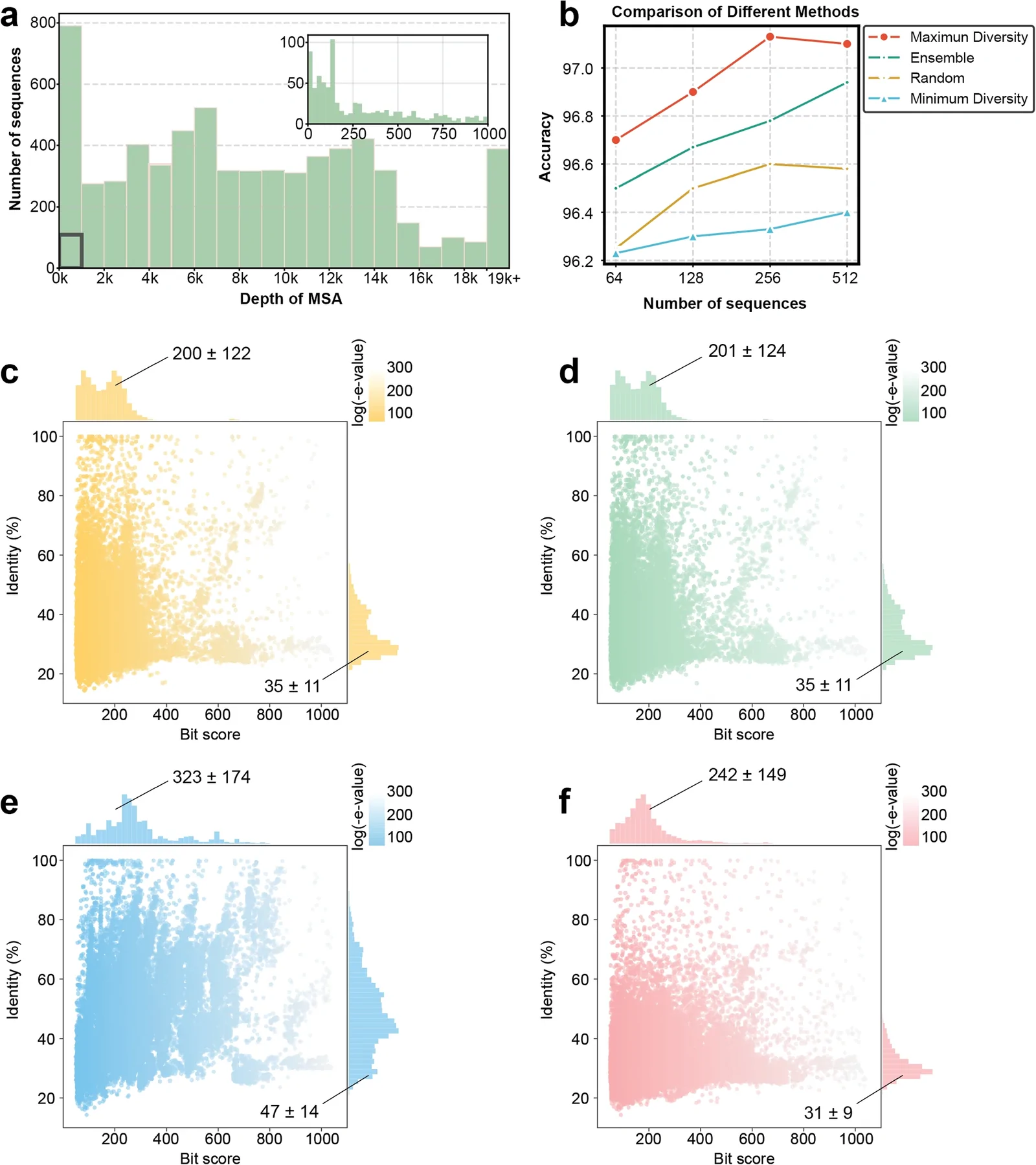
The distribution and depth of MSA and comparison of MSA selection strategies. **a** The distribution of the number of sequences in MSA. **b** Comparison of MSA selection strategies and the depth of MSA. Using maximum diversity approaches with 256 input protein sequences achieves the best performance. **c** Pairwise sequence identity vs. bit score for selected MSA by random strategy. Colors depict the exponent of the e-value. **d** Pairwise sequence identity vs. bit score for selected MSA by ensemble strategy. Colors depict the exponent of the e-value. **e** Pairwise sequence identity vs. bit score for selected MSA by minimum diversity strategy. Colors depict the exponent of the e-value. **f** Pairwise sequence identity vs. bit score for selected MSA by maximum diversity strategy. Colors depict the exponent of the e-value

In large language models, optimal memory utilization and computational resource allocation are crucial throughout the training and inference stages. Multiple Sequence Alignment consumes these resources due to its complexity. For example, given an MSA with M sequences and L length, the computational and peak memory cost of the large language model MSA Transformer is $\mathcal {O}(LM^2 +ML^2 )$. The original MSAs attain sequence depths numbering in the thousands, with the complete depth distribution presented in Fig. 4a. Therefore, effective management of memory and computational resources, particularly for MSA, is essential. This study first evaluated the performance of the primary subsampling strategies for MSA to optimize the balance between computational efficiency and model accuracy. To further identify optimal conditions and parameters for the effective subsampling deployment in practical scenarios, we then investigated how changes in the number of sequences in MSA affect model performance.

Here, we briefly introduce *four primary subsampling strategies* used in our study. *Random sampling* in the context of an MSA is a straightforward strategy where sequences are chosen randomly. This simplicity in setup and execution makes it a popular choice in various data analyses. The *ensemble approach* averages the outputs from multiple random subsamples, which can reduce the variability associated with any single random selection, although it increases computational cost. The *maximum diversity* approach employs a greedy algorithm to select sequences that show the lowest identity to those already chosen. By maximizing sequence diversity, the model potentially gains a broader exposure to varying sequences, which can enhance its generalization capabilities and improve prediction accuracy across diverse inputs. Conversely, the *minimum diversity* strategy employs a greedy algorithm to select the sequence with the highest identity to the current set, creating a similar and non-diverse MSAs to preserve sequence similarity as much as possible. This approach allows exploration of how homogeneity within sequence sets impacts model performance.

The results of our study, detailed in Fig. 4, provide clear insights into the efficacy of different subsampling strategies within the SEVA model framework. Among these strategies, maximum diversity sampling achieved the best overall performance. VFs and ARGs can contain rare but clinically significant variants [66], and therefore retaining diverse homologous sequences may help the model capture broader evolutionary variation. Random sampling and the ensemble strategy produced similar MSA search distributions, whereas the ensemble strategy showed better model performance, likely because averaging across multiple random subsamples increases coverage of the available sequence space. In contrast, although the minimum diversity strategy selected sequences with higher sequence identity and bit score than the maximum diversity strategy (Fig. 4), these selected sequences were highly redundant. Such redundancy can limit the diversity of evolutionary information and may reduce model performance. The maximum diversity strategy prioritized widely distributed sequences, which had a higher overall bit score than random and ensemble methods (Fig. 4). The bit score is a normalized alignment score that reflects the strength of sequence alignment, and a higher bit score indicates higher alignment quality between the query and reference sequences [13, 14]. These results suggest that the maximum diversity strategy can retain sequences with high alignment quality while reducing redundancy and reserving broader evolutionary information. Generally, the success of the maximum diversity strategy lies in its balance between two key requirements: retaining sequences with high alignment quality and maximizing sequence diversity.

Our study also explored the relationship between the depth of MSA and model performance (Fig. 4b). We found that increasing the depth of MSA generally enhances inference performance. However, beyond a certain point (in our study, MSA from 256 to 512 sequences), the improvement in performance is not significant. It even decreases slightly in some strategies, such as maximum diversity and random strategies. Therefore, given the trade-off between computational resources and model efficacy, we opted to subsample 256 sequences for MSA in our study. For sequences where the raw MSA depth was less than 256, the entire, original MSA without subsampling was fed into MSA Transformer to generate MSA embedding.

Besides, to better understand how MSA characteristics influence SEVA’s performance, we analyzed the impact of mean pairwise identity and the depth of MSA on the test dataset. As shown in Supplementary Fig. S11, we found that SEVA maintains high accuracy even for proteins with low mean pairwise identity. Among misclassified proteins, a greater proportion (13) came from MSAs above the median identity than from those below (5). An MSA with a high mean pairwise identity indicates that the sequences within it are relatively similar. This redundancy of information may hinder the capture of distant homologs. Additionally, the error rate was notably higher for proteins with shallow MSAs (sequences fewer than 256), at 5.17% (3 out of 58), compared to a 2.64% error rate (15 out of 569) for proteins with deep MSAs (256 or more sequences), indicating that insufficient MSA depth constrains model performance.

### Prediction of the low-identity dataset

Reliable identification of virulence factors and antibiotic resistance genes with low homology (<50%) to known reference sequences is an ongoing challenge in microbial genomics. Conventional homology-based methods, which mainly rely on pairwise sequence alignment algorithms, show significant limitations in this regard, especially when sequence homology is below 30–40%, where true remote homologs are easily missed. These limitations stem from the fundamental limitation of distinguishing true functional homologs from matches based solely on sequence similarity.

**Fig. 5:**
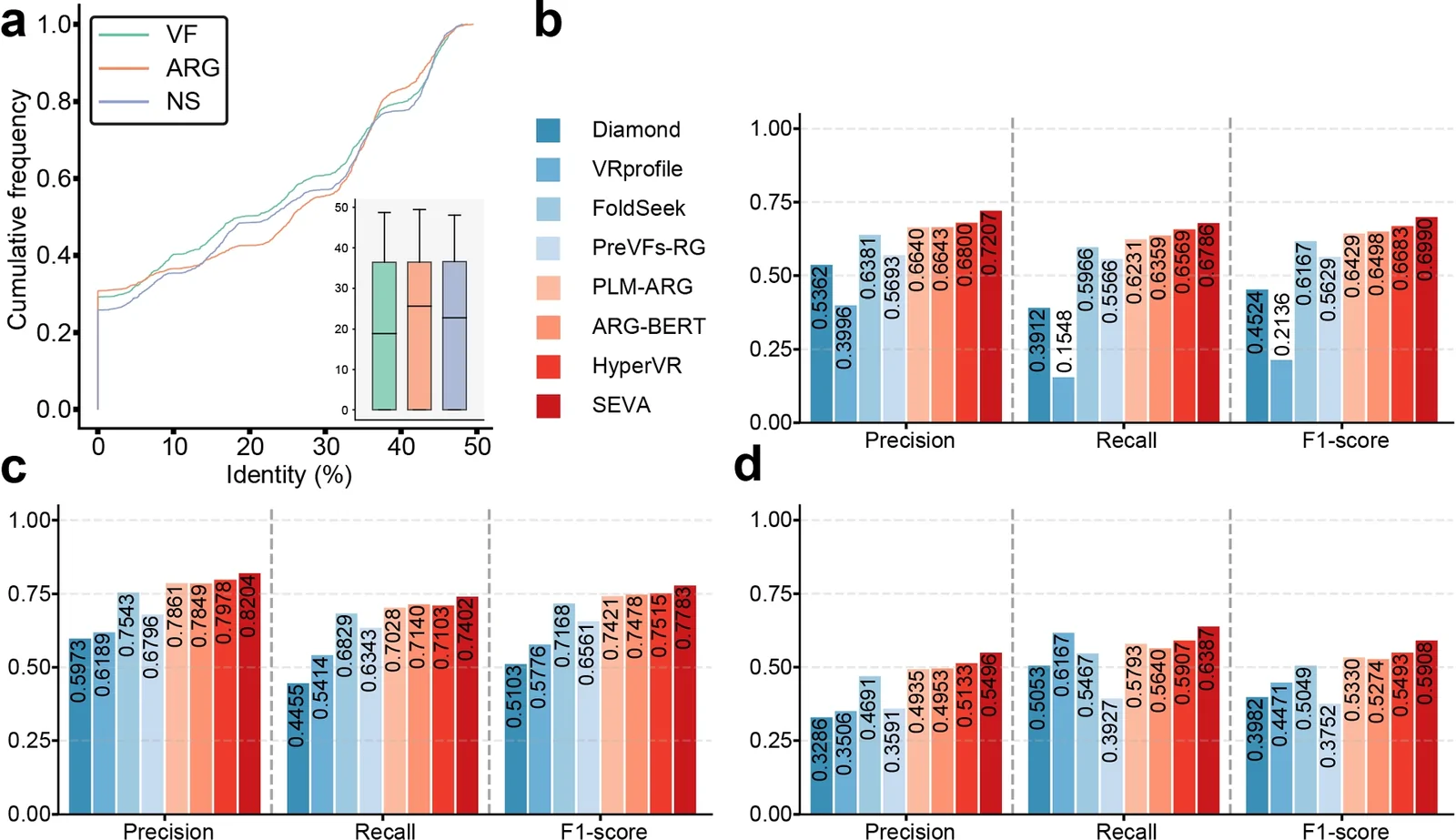
The distribution and prediction results of predicting VFs, ARGs, and NSs on the low-identity dataset. **a** The distribution of the maximum sequence identity against the training dataset and reference databases (VFDB, Victors, PATRIC, CARD, and ARDB) of the low-identity dataset. **b** The performance of VF prediction compared with Diamond, VRprofile, FoldSeek, PreVFs-RG, PLM-ARG, ARG-BERT, HyperVR, and SEVA. **c** The performance of ARG prediction compared with Diamond, VRprofile, FoldSeek, PreVFs-RG, PLM-ARG, ARG-BERT, HyperVR, and SEVA. **d** The performance of NS prediction compared with Diamond, VRprofile, FoldSeek, PreVFs-RG, PLM-ARG, ARG-BERT, HyperVR, and SEVA

To evaluate the ability of SEVA to address this critical gap, we conducted rigorous benchmarking using a dataset of 4,580 low-identity sequences containing 1475 VFs, 1605 ARGs, and 1500 NSs with <50% sequence identity against the training and reference datasets. We used eight existing tools, Diamond, VRprofile, FoldSeek, PreVFs-RG, PLM-ARG, ARG-BERT, HyperVR, and SEVA, to evaluate the performance. Notably, MSA selection strategy needs adjustment to avoid information leakage from MSA Transformer embedding on the low-identity sequences. For MSA construction of the low-identity dataset, sequences with an identity greater than 50% against any proteins in MSAs of training and reference datasets were removed using CD-HIT. The comparison results demonstrate that SEVA outperforms other approaches in remote homolog detection (Fig. 5 and Supplementary file 1: Fig. S5). For VF classification, SEVA achieved a precision of 72.07%, a recall of 67.86%, and an F1-score of 69.90%, outperforming the evaluated baseline tools. In comparison, Diamond, VRprofile, FoldSeek, PreVFs-RG, PLM-ARG, ARG-BERT, and HyperVR obtained 53.62%, 39.96%, 63.81%, 56.93%, 66.40%, 66.43%, and 68.00% precision, respectively, along with 39.12%, 15.48%, 59.66%, 55.66%, 62.31%, 63.59%, and 65.69% recall, and 45.24%, 21.36%, 61.67%, 56.29%, 64.29%, 64.98%, 66.83% F1-score. For ARG classification, SEVA demonstrated enhanced performance with 82.04% precision, representing improvements of 22.31%, 20.15%, 6.61%, 14.08%, 3.43%, 3.55%, and 2.26% over Diamond, VRprofile, FoldSeek, preVFs-RG, PLM-ARG, ARG-BERT, and HyperVR, respectively. SEVA also attained 74.02% recall and 77.83% F1-score in ARG prediction, further confirming its robustness. In NS detection, SEVA consistently outperformed all benchmarked methods, achieving 54.96% precision, 63.87% recall, and 59.08% F1-score. These results indicate that SEVA improves prediction for divergent sequences compared with existing tools, although the reduced absolute performance, especially for VF and NS prediction, shows that low-identity functional annotation remains challenging.

The enhanced performance of SEVA on the low-identity dataset can be attributed to these innovations in its computational architecture. First, integrating multiple sequence alignments enables the capture of evolutionarily conserved functional motifs. Second, the protein structure remains conserved despite the low sequence identity in some functional domains of VFs or ARGs. Third, protein language model embeddings provide robust protein representations across different evolutionary scales.

### Applying SEVA to simulated bloodstream infection samples

**Fig. 6:**
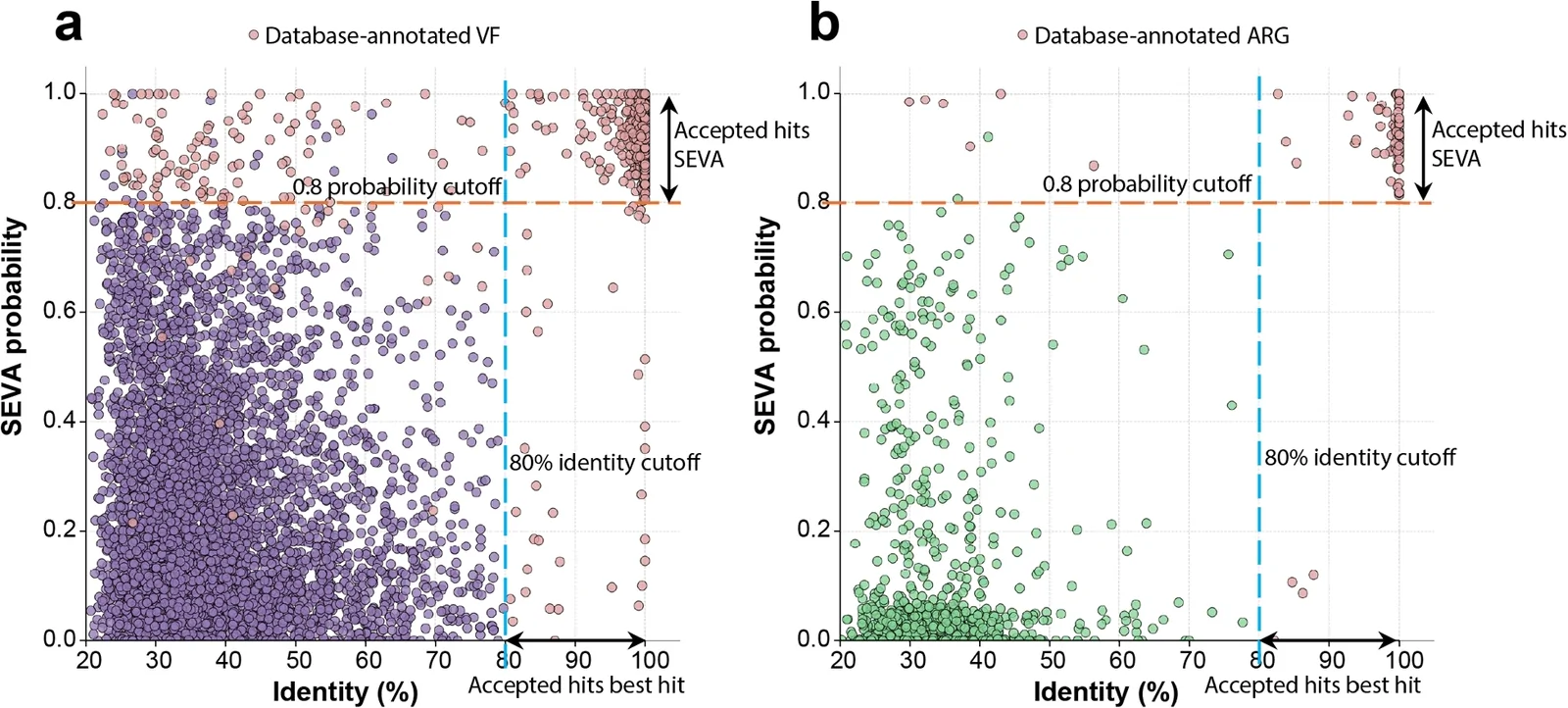
The performance on the simulated bloodstream infection samples. **a** Distribution of VF classification probability by SEVA and the best hit identity. **b** Distribution of ARG classification probability by SEVA and the best hit identity. Identity was defined as the maximum sequence identity to proteins in the training dataset and reference databases, including VFDB, Victors, PATRIC, CARD, and ARDB. The orange dashed line indicates the SEVA probability cutoff of 0.8, and the blue dashed line indicates the identity cutoff of 80%. Pink points indicate database-annotated VF or ARG targets used as reference labels in the simulated BSI dataset

Accurate identification of virulence factors and antibiotic resistance genes in metagenomic samples is important for prioritizing VF and ARG candidates in metagenomic assemblies and supporting downstream pathogen surveillance. To evaluate SEVA in a complex metagenomic background, we applied it to the simulated bloodstream infection dataset containing 15,097 coding sequences. Database-annotated reference labels for the simulated BSI dataset were assigned based on sequence and structural similarity to known VF and ARG proteins in reference databases, including VFDB, Victors, PATRIC, CARD, and ARDB. In Fig. 6, database-annotated VF and ARG targets are highlighted as pink points. SEVA used a probability cutoff of 0.8 to classify sequences as VFs or ARGs in the simulated BSI dataset. Therefore, pink points above the SEVA probability cutoff represent database-annotated VF or ARG targets classified above the cutoff by SEVA. SEVA achieved 98.97% overall accuracy on the simulated BSI dataset (Supplementary Fig. S6). For VF prediction, SEVA correctly identified 802 of 863 database-annotated VF targets, with 90.62% precision, 92.93% recall, and a 91.76% F1-score. For ARG prediction, SEVA correctly identified 124 of 128 database-annotated ARG targets, with 91.85% precision, 96.88% recall, and a 94.30% F1-score. These results indicate that SEVA can accurately classify VF, ARG, and NS proteins in a simulated metagenomic background using database-annotated reference labels.

Despite the strong overall performance, a small proportion of high identity targets were missed, including 35 VFs and 4 ARGs. We further analyzed the MSA composition of these missed high identity targets and found that many had shallow or fragmented MSAs (Supplementary file 1: Fig. S8). This indicates that these sequences have fewer homologs in existing databases, which limits the model’s ability to infer robust evolutionary features. In addition, these situations illustrate that, despite the highly sensitive SEVA model architecture, the completeness and accuracy of the underlying reference database are crucial for predictive performance. Continued curation and expansion of VFs and ARGs sequence databases are crucial to minimizing false negatives and improving the accuracy.

## Discussion

Accurate identification of virulence factors and antibiotic resistance genes holds profound implications for effective disease management and human healthcare. However, current approaches to VFs and ARGs prediction often suffer from high rates of false negatives, particularly for genes with low identity to known databases, and underutilize feature representation capabilities. Our study introduces SEVA, a deep learning-based model that utilizes large protein language models and incorporates structural and evolutionary features to enhance the classification of VFs and ARGs. Large protein language models can learn high-level functional representations and extend capabilities for sequence homology detection. Aggregating large protein language models, SEVA extracts rich biological information from raw sequencing data, enhancing its discriminative ability and adaptively capturing previously unidentified features and patterns. By integrating structural and evolutionary features, SEVA represents an important advance in microbial gene prediction, providing valuable insights into the functional characterization of VFs and ARGs. By using transfer learning, researchers can fine-tune the model for other specific targets, such as viruses or fungi.

We conducted a comprehensive comparison of the proposed SEVA model with existing tools and different features. SEVA outperformed all other methods, achieving an overall accuracy of 97.13% in predicting VF and ARG. The improved performance of SEVA can be attributed to its adoption of a more efficient and powerful algorithm, as well as its utilization of large protein language models and incorporation of structural and evolutionary features. Multiple sequence alignment captures evolutionary relationships among sequences, allowing the model to focus on conserved regions critical for gene function across diverse species. This evolutionary context enhances the ability of the model to generalize and accurately predict genes even in the presence of sequence variations or mutations. In addition, PSSM and MSA reflect the evolutionary information of protein sequences. PSSM, while benefiting from the alignments provided by MSA, focuses more on the detailed analysis of specific positions within a sequence, but MSA is primarily used for directly comparing sequences to find similarities and differences. Benefiting from the development of large language models, protein language models can be studied to perform inference from MSA. MSA reveals conserved sequences, evolutionary histories, and functional similarities, making it an essential research component to understand and detect VFs and ARGs. MSA Transformer was employed in its original form to generate the MSA embeddings used in SEVA. Besides, we discussed whether query sequence position within MSAs impacts MSA Transformer output or model performance. The results show that changing this position impacts the output embeddings of the MSA Transformer. This is likely because the model was trained with the query sequence consistently placed in the first row. Deviating from this standard causes the model to generate feature representations that may be misaligned with its training objective. Therefore, we fixed the position of the query sequence in MSAs in the first row. Moreover, by integrating structural information derived from techniques like AlphaFold2, SEVA can capture spatial constraints and interactions critical for protein function, enhancing prediction accuracy.

SEVA-Fast uses only statistical features, including AAC, DPC, DDE, PAAC, and QSO. These features capture amino acid composition, sequence-order patterns, and physicochemical properties, which can provide strong discriminative signals for many VF, ARG, and NS proteins in the benchmark dataset and may explain why SEVA-Fast achieves 91.23% accuracy. In comparison, the structure-aware alignment tool FoldSeek achieves 92.19% accuracy, and SEVA using structure-derived features alone achieves 92.50% accuracy, indicating that protein structural information is also informative for VF and ARG prediction. The apparent discrepancy is mainly due to the difference between single-feature performance and marginal improvement after feature integration. Statistical features, evolutionary features such as PSSM and MSA embeddings, and protein structural features are partially correlated and therefore not fully independent. Thus, once statistical features, PSSM features, and MSA embeddings have already been incorporated, part of the structural signal may have been indirectly captured, making the additional gain from explicit structural features smaller in absolute accuracy. Nevertheless, adding structure-derived features still improves the full model accuracy from 95.69% to 97.13%, suggesting that structural information provides a complementary signal beyond other feature types.

Notably, we observed that VF prediction is more challenging than ARG prediction, with VF and NS sequences exhibiting closer and more overlapping distributions in the latent representation space than ARG sequences. Many ARGs are associated with specialized resistance mechanisms and are frequently conserved across bacterial strains or species exposed to similar antibiotic pressures [67]. Some ARGs, such as plasmid-encoded beta-lactamases, are often carried by mobile genetic elements, enabling horizontal gene transfer between bacteria [68, 69]. Their conserved functional motifs and broad representation in ARG reference databases may make them easier to detect. In contrast, certain VFs are also involved in regular bacterial functions such as biofilm formation. Such functions can also be present in non-pathogenic bacteria or proteins annotated as negative samples, and whether they contribute to virulence depends on the host environment, strain background, genomic context, and expression regulation [70]. VFs may therefore share sequence, structural, or evolutionary features with some NS proteins, making the VF/NS boundary less distinct.

By integrating protein language model embeddings, MSA-based evolutionary representations, and structure-derived distance maps, SEVA provides a unified framework for VF and ARG prediction, with the potential to improve prediction performance and facilitate biological interpretation. However, adopting these large models requires large amounts of data, resulting in considerable time consumption and computational complexity. Balancing the need for accuracy with the practical limitations of memory requirements becomes a key consideration. To address this challenge, we emphasize the importance of employing appropriate subsampling strategies in bioinformatics applications of large protein language models. By carefully selecting and implementing a subsampling strategy, we can ensure diversity in the training data while reducing the computational burden associated with training large models. Our study demonstrates the efficacy of a maximum diversity subsampling strategy in maintaining accuracy while optimizing memory and computational efficiency, thereby emphasizing the importance of thoughtful subsampling methods in large model applications. Additionally, we discuss how MSA characteristics influence SEVA’s performance. We found that SEVA maintains high accuracy even for proteins with low mean pairwise identity, whereas relatively shallow MSAs can limit performance. Looking ahead, our study opens up several directions for future research. Firstly, there is a need to develop and refine training methodologies for large language models to enhance their efficiency and scalability. This includes exploring innovative techniques for distributed training, model compression, and transfer learning, which can help alleviate the computational burden associated with deploying large models while preserving predictive accuracy. Moreover, further investigation is needed into the efficacy of different subsampling strategies and their impact on model performance across various datasets.

Although the test set was constructed using cluster-level splitting at a 90% identity threshold, proteins in different clusters may still retain moderate sequence similarity or conserved functional domains. Therefore, performance on the test set may be higher than performance on highly divergent sequences. To evaluate generalization under a more stringent condition, we further constructed a low-identity dataset containing sequences with less than 50% identity to the training and reference datasets. SEVA maintained the best overall performance among the evaluated methods on the low-identity dataset, supporting its improved generalization to divergent sequences. Nevertheless, performance decreases on the low-identity dataset compared with the test set, indicating that although SEVA improves low-identity prediction compared with existing methods, distant homology prediction remains challenging. We also evaluated the performance of SEVA on simulated bloodstream infection samples. Furthermore, the simulated BSI analysis supports the potential use of SEVA for prioritizing VF and ARG candidates in complex metagenomic backgrounds, while further validation with experimentally confirmed clinical samples will be needed before diagnostic application.

We developed SEVA-Fast, a lightweight version of SEVA that omits the MSA search, PSSM generation, and structure prediction steps and relies only on statistical features. SEVA-Fast markedly reduces runtime and memory requirements, making it suitable for large-scale preliminary screening. On the test dataset, SEVA-Fast retained high overall accuracy despite performing below full SEVA, suggesting that statistical features capture useful signals for discriminating many VFs, ARGs, and NSs. On the simulated BSI dataset, SEVA-Fast also achieved high overall accuracy, although its VF and ARG class-level F1-scores were lower than those of full SEVA. On the independent ARG test dataset, SEVA-Fast performed close to full SEVA, consistent with the relatively distinct and conserved sequence patterns of many ARGs. By contrast, the performance gap was larger on the low-identity dataset (Supplementary file 1: Table S10), suggesting that statistical features alone are less sufficient for distant sequences and that the additional evolutionary information and structural features incorporated in full SEVA may provide complementary benefits. Therefore, SEVA-Fast is best suited for rapid preliminary screening, whereas full SEVA should be preferred when maximum predictive reliability is required.

Although SEVA improves VF and ARG prediction, several limitations remain. (1) Limited training data. The performance of computational methods depends heavily on the quality and diversity of training data. Although high-quality databases were used in this study, the available training data for VFs and ARGs remain limited. Continued expansion and curation of VF, ARG, and NS databases through community efforts will be important for improving model accuracy and generalization. (2) Limitations of negative sample collection. The NS dataset comprises proteins with no known homology to the VF and ARG databases for performance evaluation. However, metagenomic applications often involve hypothetical, poorly annotated, or functionally ambiguous proteins. These proteins may introduce additional uncertainty in distinguishing VF, ARG, and NS categories and may increase the risk of false-positive VF or ARG predictions in practical use. Incorporating more diverse and realistically sampled background proteins in future work may further improve the robustness and practical reliability of SEVA. (3) Computational cost. The full SEVA pipeline requires MSA construction and structure prediction, which increases runtime and memory requirements. Although SEVA-Fast provides a lightweight alternative for rapid screening, full SEVA remains computationally demanding when applied to large metagenomic datasets. (4) Low-identity prediction remains challenging. Although SEVA outperformed existing methods on the low-identity dataset, its performance was lower than on the test dataset, indicating that distant homolog detection remains challenging.

## Conclusions

SEVA is a deep learning framework that integrates protein large language model embeddings with evolutionary and structural features to predict virulence factors and antibiotic resistance genes. This integration enables SEVA to learn rich functional representations from protein sequence, evolutionary patterns, and structural information. Comprehensive evaluation shows that SEVA outperforms the evaluated baseline methods on the test set, improves prediction on low-identity sequences compared with existing tools, recovers most database-annotated VF and ARG candidates in the simulated BSI metagenomic dataset, and performs well on the independent ARG dataset. These findings demonstrate that combining evolutionary and structural information can better identify functionally diverse microbial genes, especially those that are difficult to detect based on sequence similarity alone. Furthermore, SEVA-Fast provides a lightweight, rapid, and large-scale screening protocol when computational efficiency is prioritized. In conclusion, SEVA offers an accurate and flexible framework for predicting virulence factors and antibiotic resistance genes, with potential applications in pathogen surveillance, antimicrobial resistance monitoring, and metagenomic functional annotation.

## Supplementary Information

Supplementary Material 1.

## Data Availability

The datasets and source code supporting the conclusions of this article are available at https://github.com/kaiqili2/SEVA and Zenodo at https://doi.org/10.5281/zenodo.17168354.
