# Supplementary material for: SEVA: structural and evolutionary feature integration for predicting virulence factors and antibiotic resistance genes
Source: Microbiome. 2026 Jul 18;14:230. doi: 10.1186/s40168-026-02467-w (PMC13660298; doi:10.1186/s40168-026-02467-w)
Supplement: Supplementary file 1 [file 40168_2026_2467_MOESM1_ESM.pdf]

Supplementary Figures

Algorithm 1 Maximum Diversity Selection

Require: Set of protein sequences $S = \{s_1, s_2, \ldots, s_N\}$, target subset size len $= 256$

Ensure: Diverse subset $S_{\text{selected}} \subseteq S$ where $|S_{\text{selected}}| = \text{len}$

$S_{\text{selected}} \leftarrow \{s_1\}$

while $|S_{\text{selected}}| < \text{len}$ **do**

$$s^* \leftarrow \arg\min_{s \in S \setminus S_{\text{selected}}} \left(\max_{s' \in S_{\text{selected}}} \text{Identity}(s, s') \right)$$

$S_{\text{selected}} \leftarrow S_{\text{selected}} \cup \{s^*\}$

end while

return S_{selected}

Fig. S1. The algorithm of maximum diversity MSA selection. The algorithm iteratively selects the sequence with the lowest pairwise identity to the current chosen set until the target subset size is reached.

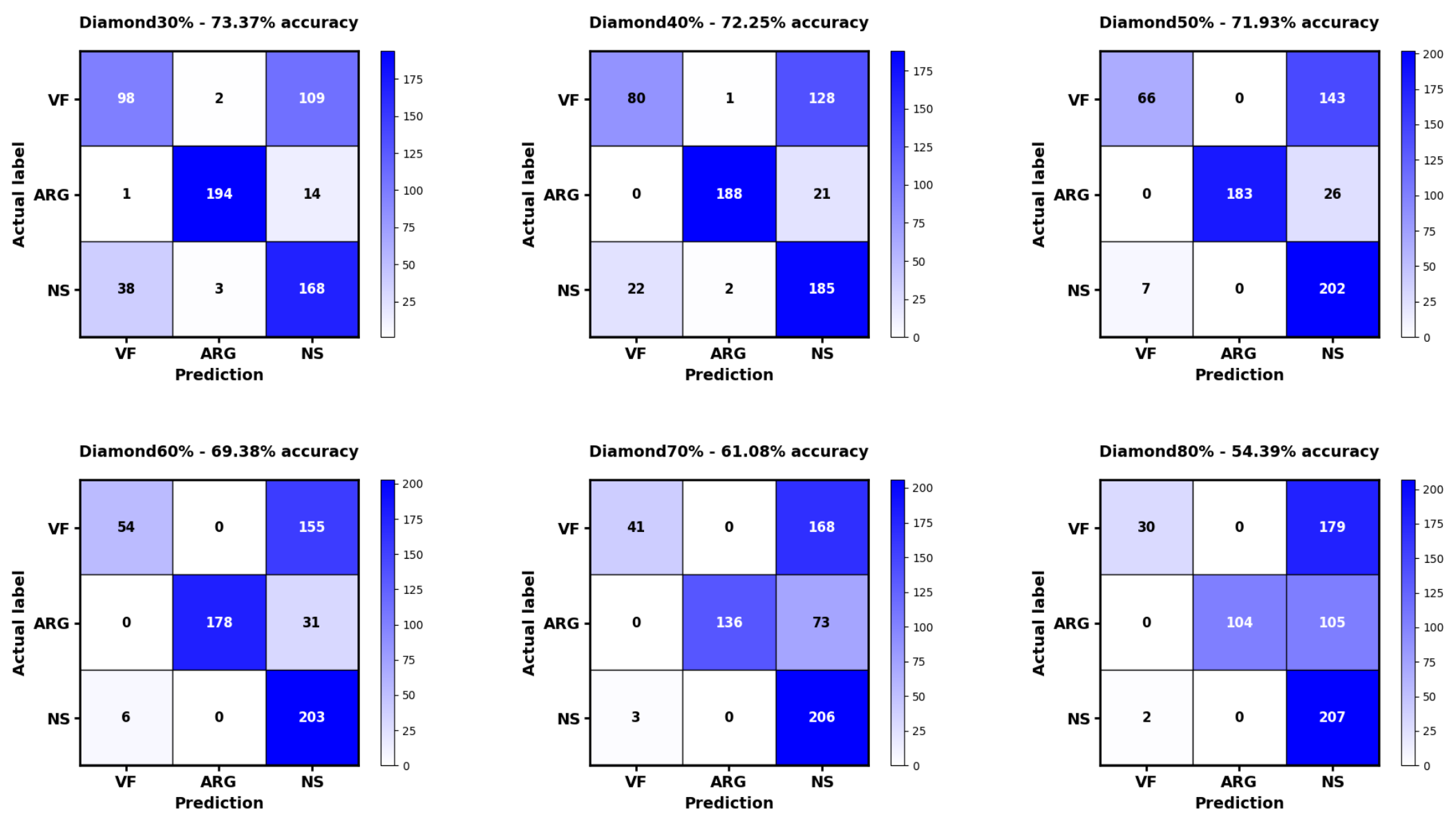

Fig. S2. Performance on the test dataset using the Diamond homology search method with different identity thresholds. As the identity threshold decreases, the Diamond algorithm achieves higher accuracy on the test dataset. Diamond with a 30% identity threshold achieves 73.37% accuracy.

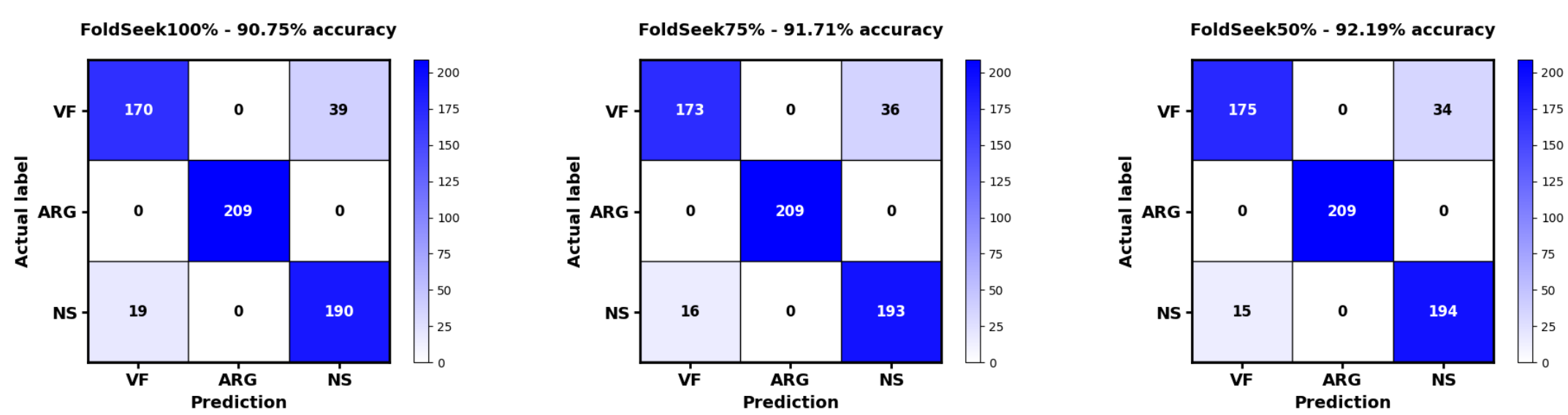

Fig. S3. Performance on the test dataset using FoldSeek with different probability thresholds. FoldSeek with a 50% probability threshold achieves 92.19% accuracy.

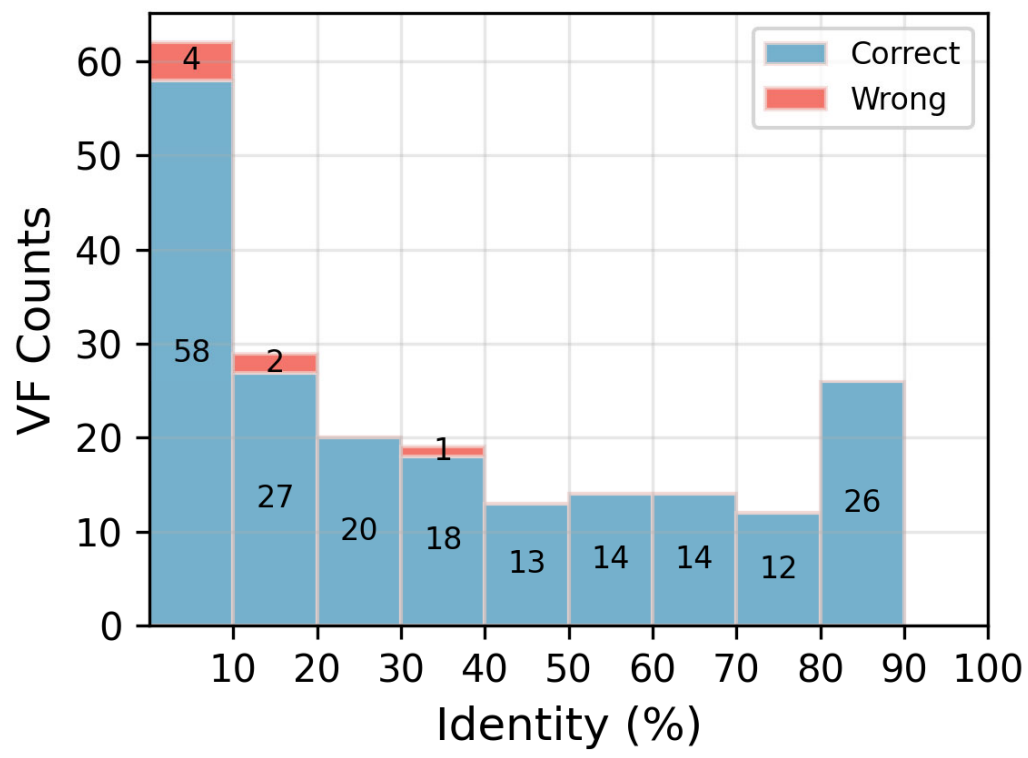

Fig. S4. Analysis of misclassified virulence factors on the test dataset. All false-negative predictions correspond to sequences with low pairwise identity (<40%) to those in the training dataset.

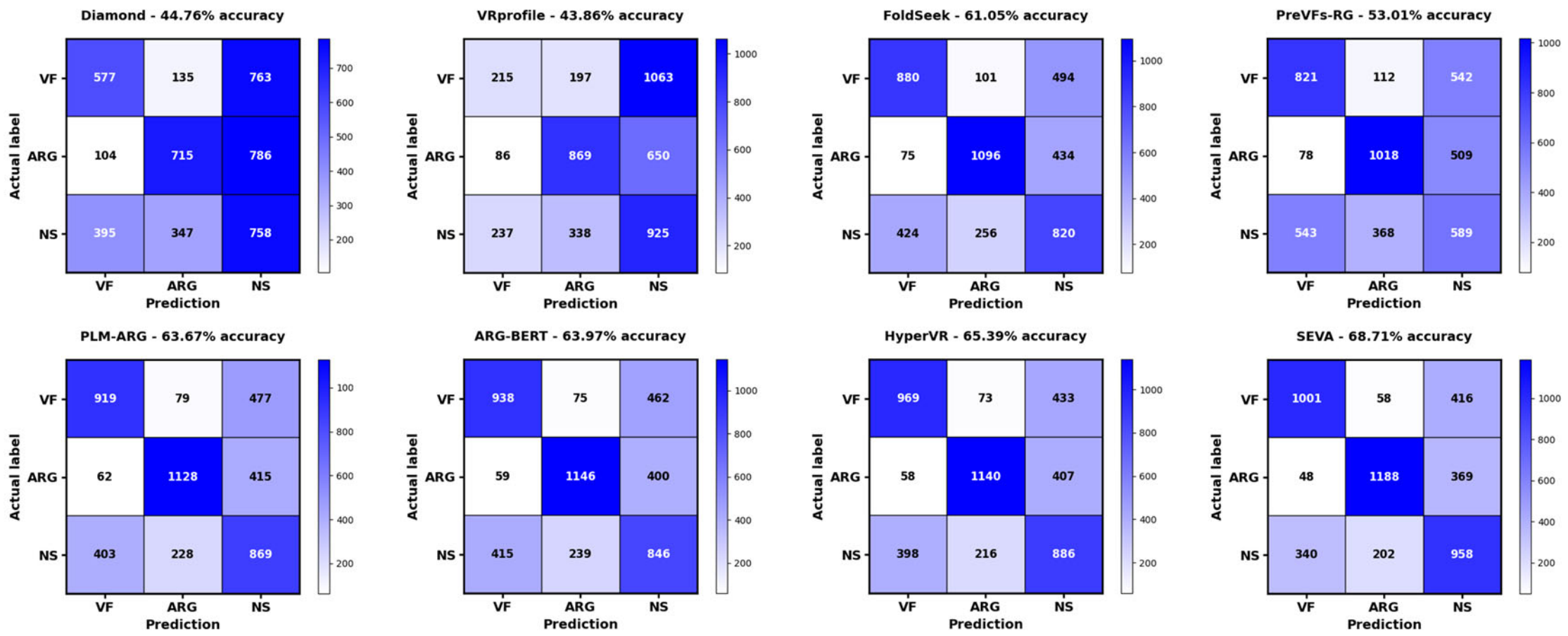

Fig. S5. Comparative performance of eight methods on the low-identity dataset. The confusion matrix reveals that SEVA outperforms Diamond, VRprofile, FoldSeek, PreVFs-RG, PLM-ARG, ARG-BERT, and HyperVR on the low-identity dataset, achieving 68.71% accuracy.

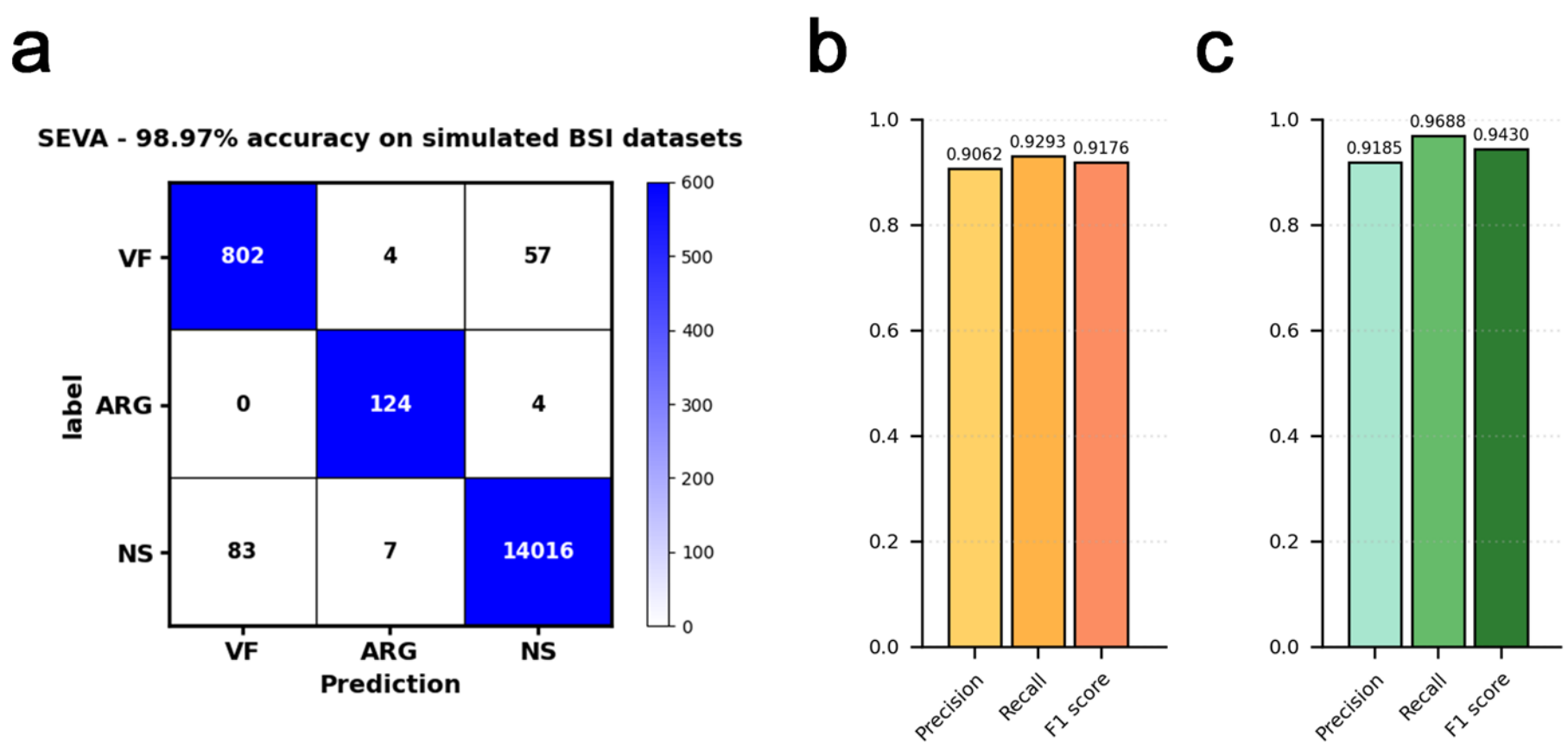

Fig. S6. Performance on the simulated bloodstream infection dataset. (a) The confusion matrix of SEVA achieves 98.97% accuracy. (b) The precision, recall, and F1-score of VF prediction on the simulated bloodstream infection dataset. (c) The precision, recall, and F1-score of ARG prediction on the simulated bloodstream infection dataset. Labels were assigned using database annotation, based on sequence and structural similarity to known VF and ARG proteins in reference databases including VFDB, Victors, PATRIC, CARD, and ARDB.

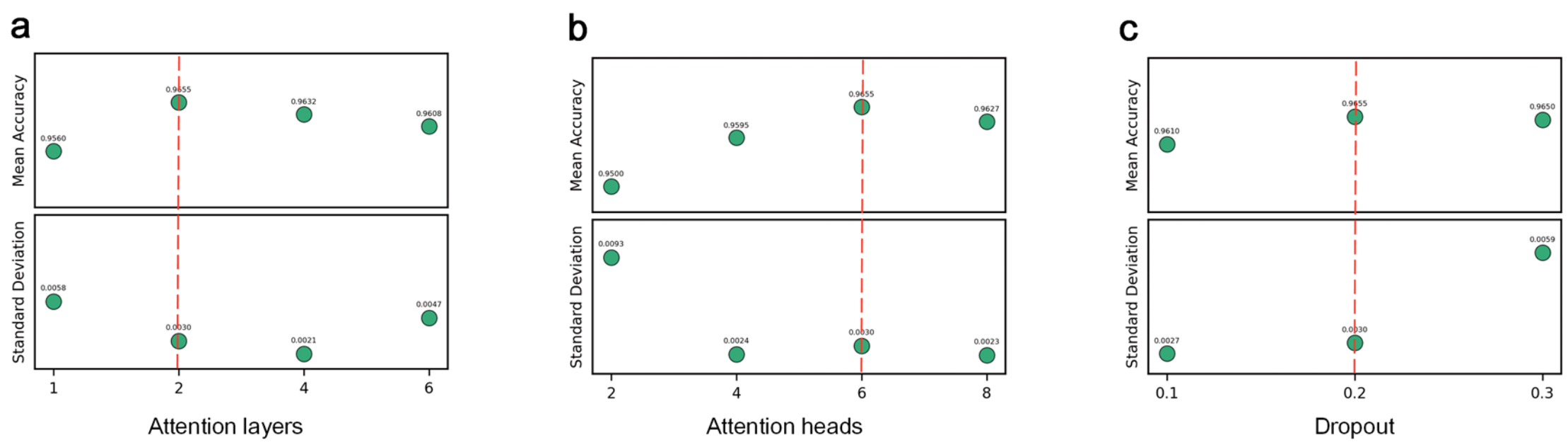

Fig. S7. Hyperparameter optimization for the SEVA development. (a) Distributions of mean and standard deviations of accuracy of SEVA on five-fold cross-validation with different *attention layers*. (b) Distributions of mean and standard deviations of accuracy of SEVA on five-fold cross-validation with different *attention heads*. (c) Distributions of mean and standard deviations of accuracy of SEVA on five-fold cross-validation with different *dropout rates.*

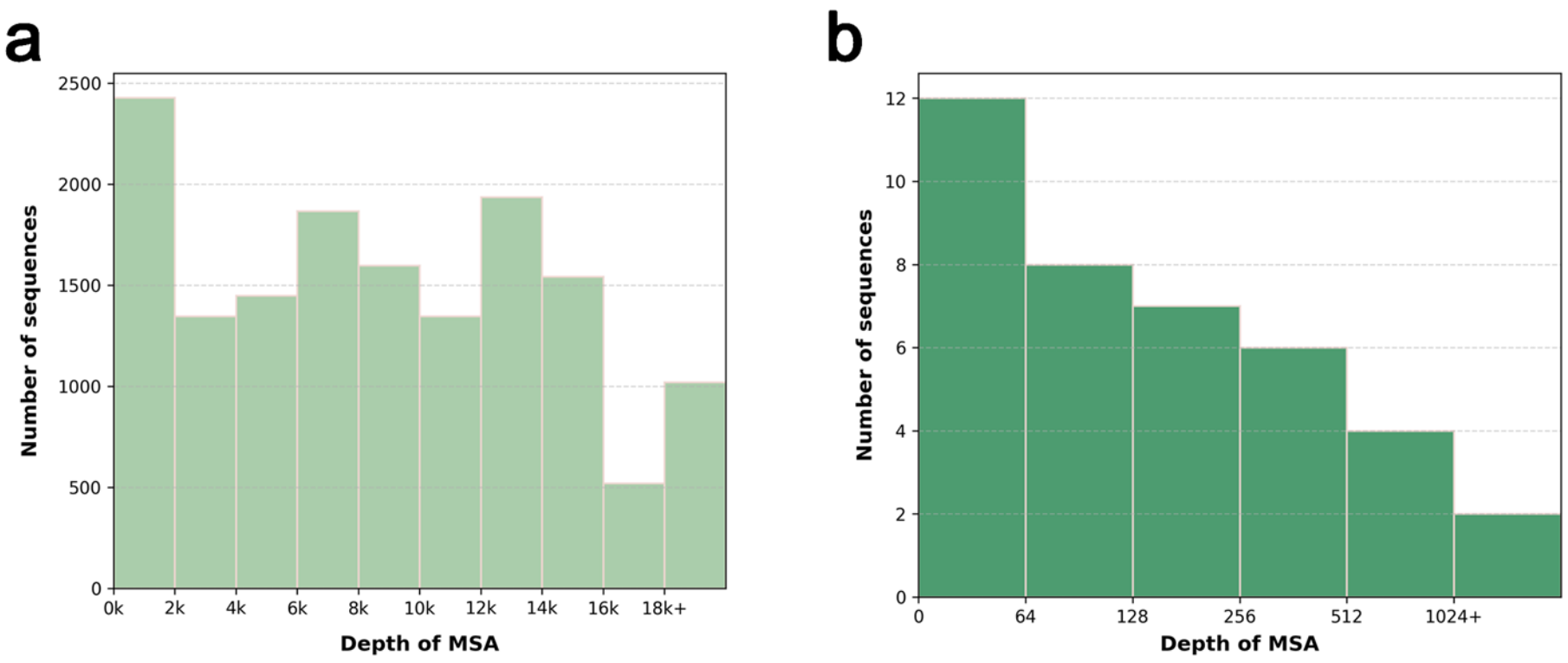

Fig. S8. Analysis of missed high-identity targets on the simulated bloodstream infection dataset. (a) Distribution of MSA depths for all targets. (b) Distribution of MSA depths for the missed high-identity targets. The majority of missed targets have shallow MSAs (fewer than 256 sequences).

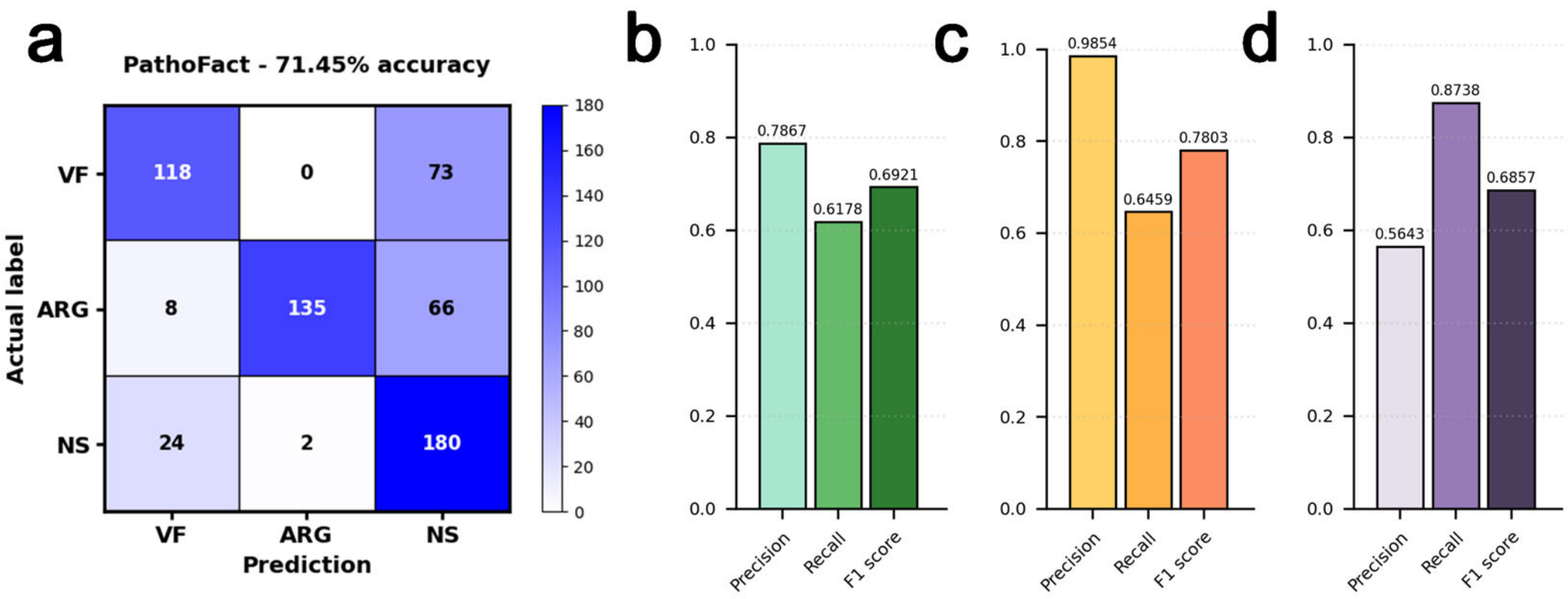

Fig. S9. Performance of PathoFact on the test dataset. (a) The confusion matrix of PathoFact achieves 71.45% accuracy. (b–d) Precision, recall, and F1-score for the prediction of (b) virulence factors, (c) antibiotic resistance genes, and (d) negative samples.

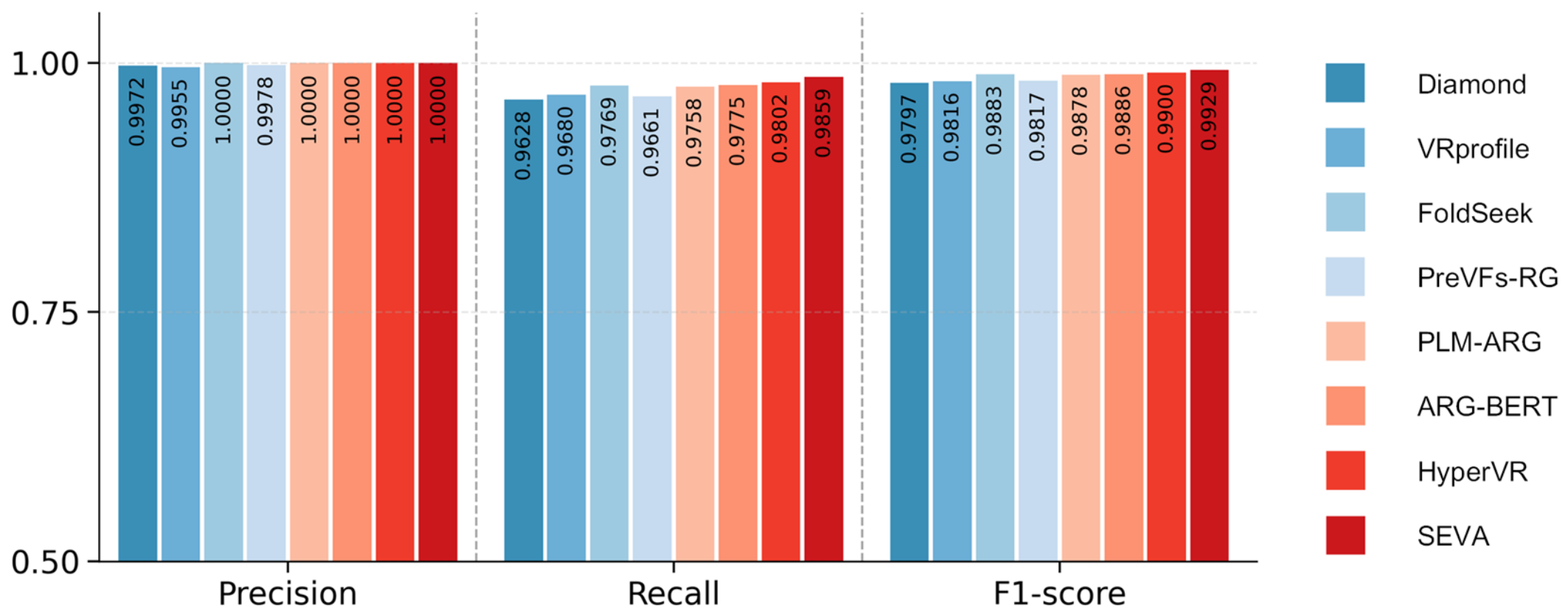

Fig. S10. Performance on the independent ARG test dataset. SEVA outperforms Diamond, VRprofile, FoldSeek, PreVFs-RG, PLM-ARG, ARG-BERT, and HyperVR on the independent ARG test dataset, achieving 100% precision, 98.59% recall, and 99.29% F1-score.

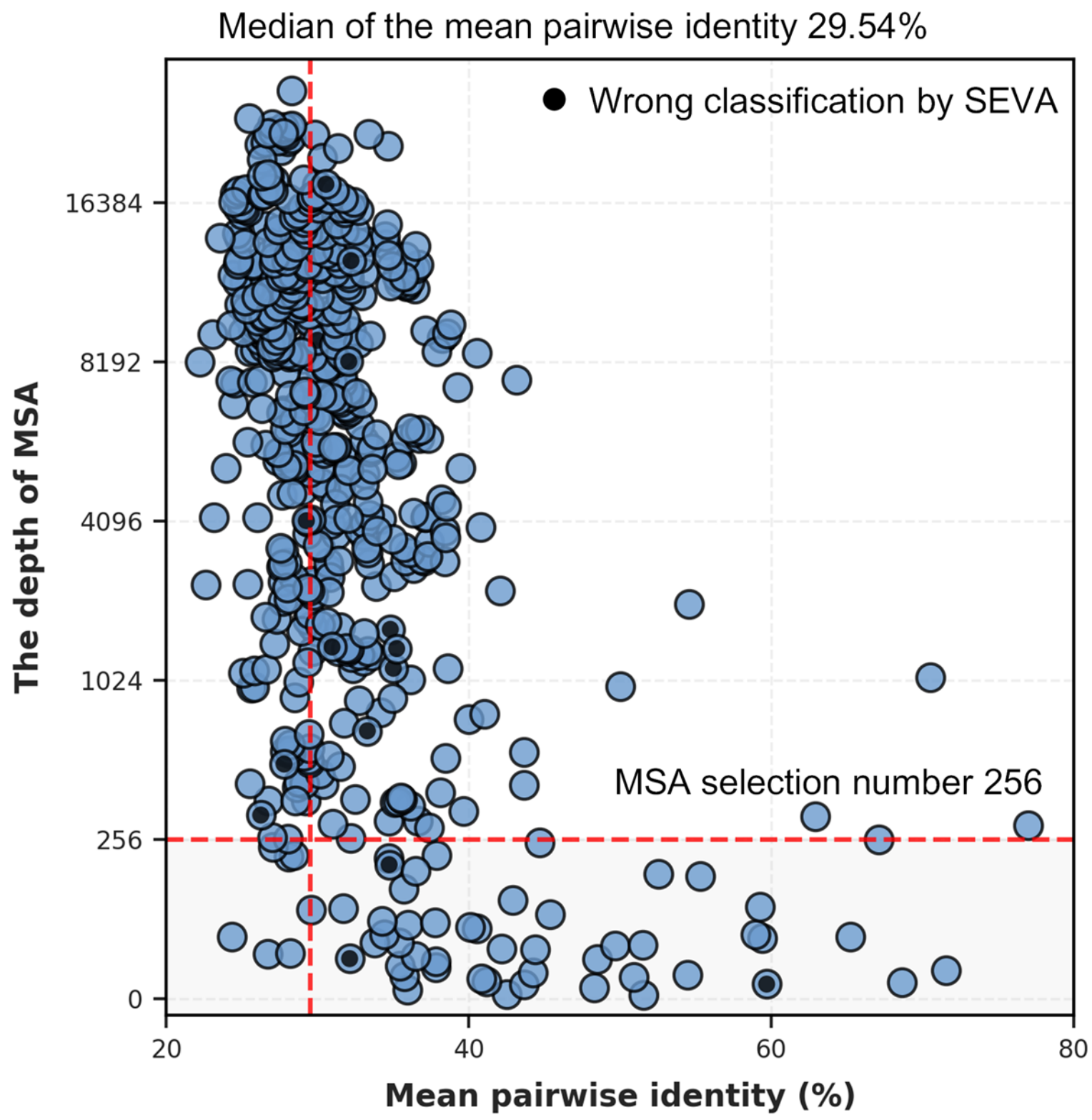

Fig. S11. Analysis of SEVA performance with MSA depth and mean pairwise identity. This scatter plot shows the distribution of original multiple sequence alignment depth and average pairwise sequence identity after maximum diversity selection. Each blue circle represents a protein. Circles with black dots indicate cases where the SEVA method caused classification errors.

Supplementary Tables

Table S1. The network and parameters of the proposed SEVA. The Adam optimizer was utilized with a learning rate of 1e-4 and trained with a batch size of 32 for 50 epochs.

Component	Layer	Parameter
Transformer	SelfAttention	dim = 768, heads = 6, dropout = 0.2
	FeedForwardNetwork	dropout = 0.2
	SelfAttention	dim = 768, heads = 6, dropout = 0.2
	FeedForwardNetwork	dropout = 0.2
Classification	LayerNorm	dim = 768
	Linear	kernel number = 2922, 512
	Linear	kernel number = 512, 256
	Linear	kernel number = 256, 3

Table S2. Detailed information on statistical and PSSM features.

	Feature	Size	Description
Statistical features	AAC	20	The proportion of each of the 20 standard amino acids in a protein sequence.
	DPC	400	The frequency of dipeptides in a protein sequence.
	DDE	400	The dipeptide deviation from the expected mean.
	PAAC	50	The pseudo-amino acid composition. Extends AAC by incorporating sequence-order effects through correlation factors.
	QSO	64	The quasi-sequence order. Similar to PAAC, it uses a distance matrix of amino acids.
PSSM features	PSSM composition	400	The PSSM composition averages all rows of PSSM.
	RPM-PSSM	400	The RPM-PSSM transforms the original PSSM by filtering the negative values to 0.
	AADP-PSSM	420	The AADP-PSSM extends the traditional AAC and DPC to PSSM.

Table S3. Comparative performance with different protein language models on the five-fold cross-validation. Five-fold cross-validation using three protein language models: ESM-2, ProtT5, and MSA Transformer. Except for the protein language model, other components are the same as those of SEVA.

		Precision	Recall	F1-score	Accuracy
ESM-2	VFs	0.9523 ± 0.0099	0.9282 ± 0.0080	0.9400 ± 0.0025	0.9573 ± 0.0024
	ARGs	0.9950 ± 0.0002	0.9905 ± 0.0009	0.9927 ± 0.0004	
	NSs	0.9258 ± 0.0092	0.9535 ± 0.0097	0.9394 ± 0.0040	
ProtT5	VFs	0.9531 ± 0.0059	0.9303 ± 0.0051	0.9415 ± 0.0030	0.9595 ± 0.0024
	ARGs	0.9950 ± 0.0002	0.9939 ± 0.0022	0.9945 ± 0.0012	
	NSs	0.9311 ± 0.0027	0.9540 ± 0.0069	0.9424 ± 0.0038	
MSA Transformer	VFs	0.9656 ± 0.0029	0.9371 ± 0.0073	0.9511 ± 0.0035	0.9655 ± 0.0030
	ARGs	0.9975 ± 0.0001	0.9930 ± 0.0017	0.9953 ± 0.0008	
	NSs	0.9347 ± 0.0084	0.9665 ± 0.0042	0.9503 ± 0.0051	

Table S4. Comparative performance with different methods on the five-fold cross-validation. Five-fold cross-validation with five machine learning methods: PreVFs-RG, PLM-ARG, ARG-BERT, HyperVR, and SEVA.

		Precision	Recall	F1-score	Accuracy
PreVFs-RG	VFs	0.7844 ± 0.0163	0.7890 ± 0.0116	0.7867 ± 0.0121	0.8288 ± 0.0100
	ARGs	0.9614 ± 0.0014	0.9230 ± 0.0089	0.9418 ± 0.0047	
	NSs	0.7488 ± 0.0208	0.7745 ± 0.0125	0.7613 ± 0.0149	
PLM-ARG	VFs	0.9405 ± 0.0041	0.8155 ± 0.0059	0.8736 ± 0.0045	0.9172± 0.0040
	ARGs	0.9840 ± 0.0017	0.9900 ± 0.0004	0.9870 ± 0.0010	
	NSs	0.8394 ± 0.0085	0.9460 ± 0.0037	0.8895 ± 0.0059	
ARG-BERT	VFs	0.9364 ± 0.0077	0.8198 ± 0.0066	0.8742 ± 0.0069	0.9175± 0.0024
	ARGs	0.9851 ± 0.0006	0.9904 ± 0.0022	0.9877 ± 0.0014	
	NSs	0.8418 ± 0.0050	0.9420 ± 0.0046	0.8891 ± 0.0035	
HyperVR	VFs	0.9371 ± 0.0057	0.8412 ± 0.0083	0.8866 ± 0.0054	0.9250± 0.0049
	ARGs	0.9909 ± 0.0015	0.9906 ± 0.0015	0.9907 ± 0.0005	
	NSs	0.8554 ± 0.0096	0.9435 ± 0.0056	0.8973 ± 0.0064	
SEVA	VFs	0.9656 ± 0.0029	0.9371 ± 0.0073	0.9511 ± 0.0035	0.9655 ± 0.0030
	ARGs	0.9975 ± 0.0001	0.9930 ± 0.0017	0.9953 ± 0.0008	
	NSs	0.9347 ± 0.0084	0.9665 ± 0.0042	0.9503 ± 0.0051	

Table S5. The performance of SEVA on the five-fold cross-validation using different hyperparameters.

Hyperparameter	Value	Five-Fold Cross-Validation Accuracy
Layers	1	0.9560 ± 0.0058
	2	0.9655 ± 0.0030
	4	0.9632 ± 0.0021
	6	0.9608 ± 0.0047
Heads	2	0.9500 ± 0.0093
	4	0.9595 ± 0.0024
	6	0.9655 ± 0.0030
	8	0.9627 ± 0.0023
Dropout	0.1	0.9610 ± 0.0027
	0.2	0.9655 ± 0.0030
	0.3	0.9650 ± 0.0059

Table S6. Performance comparison of different CD-HIT thresholds to split training and test datasets.

CD-HIT threshold		Precision	Recall	F1-score	Accuracy
1.0	VFs	0.9706	0.9474	0.9588	0.9729
	ARGs	1.0000	1.0000	1.0000	
	NSs	0.9486	0.9713	0.9598	
0.9	VFs	0.9659	0.9474	0.9565	0.9713
	ARGs	1.0000	1.0000	1.0000	
	NSs	0.9484	0.9665	0.9573	
0.8	VFs	0.9655	0.9378	0.9515	0.9681
	ARGs	1.0000	1.0000	1.0000	
	NSs	0.9395	0.9665	0.9528	
0.7	VFs	0.9559	0.9330	0.9443	0.9633
	ARGs	1.0000	1.0000	1.0000	
	NSs	0.9346	0.9569	0.9456	
0.6	VFs	0.9510	0.9282	0.9395	0.9601
	ARGs	1.0000	1.0000	1.0000	
	NSs	0.9299	0.9522	0.9409	
0.5	VFs	0.9455	0.9139	0.9294	0.9537
	ARGs	0.9952	1.0000	0.9976	
	NSs	0.9209	0.9474	0.9340	
0.4	VFs	0.9453	0.9091	0.9268	0.9490
	ARGs	0.9952	0.9904	0.9928	
	NSs	0.9083	0.9474	0.9274	

Table S7. Comparative performance on the test dataset with statistical features, PSSM, MSA, and protein structure individually.

		Precision	Recall	F1-score	Accuracy
Statistical Features	VFs	0.9149	0.8230	0.8665	0.9123
	ARGs	0.9905	1.0000	0.9952	
	NSs	0.8377	0.9139	0.8741	
PSSM	VFs	0.9158	0.8325	0.8722	0.9155
	ARGs	0.9905	1.0000	0.9952	
	NSs	0.8451	0.9139	0.8782	
MSA	VFs	0.9162	0.8373	0.8750	0.9171
	ARGs	0.9858	1.0000	0.9929	
	NSs	0.8527	0.9139	0.8822	
Protein Structure	VFs	0.9271	0.8571	0.8878	0.9250
	ARGs	0.9858	1.0000	0.9929	
	NSs	0.8655	0.9234	0.8935	

Table S8. Selected complete genome sequences from NCBI.

Accession ID	Species
NC_011586.2	*Acinetobacter baumannii*
NC_000964.3	*Bacillus subtilis*
NC_001318.1	*Borreliella burgdorferi*
NC_007511.1	*Burkholderia lata*
NC_020291.1	*Clostridium saccharoperbutylacetonicum*
NC_014500.1	*Dickeya dadantii*
NC_014121.1	*Enterobacter cloacae*
NC_004668.1	*Enterococcus faecalis*
NC_000913.3	*Escherichia coli*
NC_015663.1	*Klebsiella aerogenes*
NC_014376.1	*Lacrimispora saccharolytica*
NC_007576.1	*Latilactobacillus sakei*
NC_003210.1	*Listeria monocytogenes*
NC_011757.1	*Methylorubrum extorquens*
NC_010612.1	*Mycobacterium marinum*
NC_008596.1	*Mycolicibacterium smegmatis*
NC_008767.1	*Neisseria meningitidis*
NC_004547.2	*Pectobacterium atrosepticum*
NC_010729.1	*Porphyromonas gingivalis*
NC_010554.1	*Proteus mirabilis*
NC_002516.2	*Pseudomonas aeruginosa*
NC_003197.2	*Salmonella enterica*
NC_045512.2	*SARS-CoV-2*
NC_020064.1	*Serratia*
NC_004337.2	*Shigella flexneri*
NC_007712.1	*Sodalis glossinidius*
NC_002758.2	*Staphylococcus aureus*
NC_004350.2	*Streptococcus mutans*
NC_002505.1	*Vibrio cholerae*
NC_003143.1	*Yersinia pestis*

Table S9. Performance of SEVA-Fast on the test, low-identity, simulated bloodstream infection, and independent ARG test datasets.

Dataset		Precision	Recall	F1-score	Accuracy
Test dataset	VFs	0.9149	0.8230	0.8665	0.9123
	ARGs	0.9905	1.0000	0.9952	
	NSs	0.8377	0.9139	0.8741	
Low-identity dataset	VFs	0.6086	0.5661	0.5866	0.5821
	ARGs	0.7351	0.6623	0.6968	
	NSs	0.4359	0.5120	0.4709	
Simulated bloodstream infection dataset	VFs	0.6883	0.8494	0.7604	0.9669
	ARGs	0.7469	0.9453	0.8345	
	NSs	0.9910	0.9743	0.9826	
Independent ARG test dataset	ARGs	0.9986	0.9701	0.9842	0.9701

Table S10. Comparison of full SEVA and SEVA-Fast in terms of features, computational cost, and prediction performance.

		SEVA	SEVA-Fast
Features		Statistical features, PSSM, MSA, and protein structure	Statistical features only
Average runtime per sequence		Approximately 1070 seconds	Approximately 0.36 seconds
Peak CPU RAM		48.6 GB	3.6 GB
Peak GPU memory		26.3 GB	2.8 GB
Accuracy	Test dataset	97.13%	91.23%
	Low-identity dataset	68.71%	58.21%
	Simulated BSI dataset	98.97%	96.69%
	Independent ARG test dataset	98.59%	97.01%
Recommended use case		Maximum accuracy	Rapid screening of large datasets
